# Transcriptomic, Proteomic, and Genomic Mutational Fraction Differences Based on HPV Status Observed in Patient-Derived Xenograft Models of Penile Squamous Cell Carcinoma

**DOI:** 10.3390/cancers16051066

**Published:** 2024-03-06

**Authors:** Niki M. Zacharias, Luis Segarra, Keiko Akagi, Natalie Wall Fowlkes, Huiqin Chen, Angelita Alaniz, Carolyn de la Cerda, Pedro Pesquera, Yuanxin Xi, Jing Wang, Jad Chahoud, Xin Lu, Priya Rao, Magaly Martinez-Ferrer, Curtis A. Pettaway

**Affiliations:** 1 Department of Urology, University of Texas MD Anderson Cancer Center, Houston, TX 77030, USA; lasegarra@mdanderson.org (L.S.); pipesquera@mdanderson.org (P.P.); 2 MD Anderson UTHealth Graduate School, Houston, TX 77030, USA; 3 Department of Thoracic Head & Neck Medical Oncology, MD Anderson Cancer Center, Houston, TX 77030, USA; kakagi@mdanderson.org; 4 Department of Veterinary Medicine & Surgery, MD Anderson Cancer Center, Houston, TX 77030, USA; nwfowlkes@mdanderson.org; 5 Biostatistics, University of Texas MD Anderson Cancer Center, Houston, TX 77030, USA; hchen1@mdanderson.org; 6 Center for Health Promotion and Prevention Research, University of Texas Health Science Center at Houston, Houston, TX 77030, USA; angelita.alaniz@uth.tmc.edu; 7 Department of Surgical Oncology, MD Anderson Cancer Center, Houston, TX 77030, USA; cade1@mdanderson.org; 8 Department of Bioinformatics and Computational Biology, MD Anderson Cancer Center, Houston, TX 77030, USA; yxi@mdanderson.org (Y.X.); jingwang@mdanderson.org (J.W.); 9 Department of Genitourinary Oncology, H. Lee Moffitt Cancer Center and Research Institute, Tampa, FL 33612, USA; jad.chahoud@moffitt.org; 10 Department of Biological Sciences, University of Notre Dame, Norte Dame, IN 46556, USA; xlu@nd.edu; 11 Department of Pathology, University of Texas MD Anderson Cancer Center, Houston, TX 77030, USA; prao@mdanderson.org; 12 Department of Pharmaceutical Sciences, University of Puerto Rico Medical Sciences Campus & Cancer Biology, UPR Comprehensive Cancer Center, San Juan, PR 00936, USA; magaly.martinez@upr.edu

**Keywords:** penile squamous cell carcinoma, human papillomavirus-positive penile squamous cell carcinoma, APOBEC mutations, patient-derived xenograft

## Abstract

**Simple Summary:**

Penile cancer is a rare but aggressive cancer. After it metastasizes, the median survival time is less than 12 months. The overall response rate to common first-line combination chemotherapy treatments is approximately 50%. There is an urgent need in advanced-penile-cancer treatment to find novel therapies that would generate better response rates than standard chemotherapy thus far and have less toxicity. Partially due to its rarity, there are few animal models and cell lines of penile cancer. We report on the generation of seven penile cancer animal models that were created by directly implanting human tumor tissue into immunocompromised mice.

**Abstract:**

Metastatic penile squamous cell carcinoma (PSCC) has only a 50% response rate to first-line combination chemotherapies and there are currently no targeted-therapy approaches. Therefore, we have an urgent need in advanced-PSCC treatment to find novel therapies. Approximately half of all PSCC cases are positive for high-risk human papillomavirus (HR-HPV). Our objective was to generate HPV-positive (HPV+) and HPV-negative (HPV−) patient-derived xenograft (PDX) models and to determine the biological differences between HPV+ and HPV− disease. We generated four HPV+ and three HPV− PSCC PDX animal models by directly implanting resected patient tumor tissue into immunocompromised mice. PDX tumor tissue was found to be similar to patient tumor tissue (donor tissue) by histology and short tandem repeat fingerprinting. DNA mutations were mostly preserved in PDX tissues and similar APOBEC (apolipoprotein B mRNA editing catalytic polypeptide) mutational fractions in donor tissue and PDX tissues were noted. A higher APOBEC mutational fraction was found in HPV+ versus HPV− PDX tissues (*p* = 0.044), and significant transcriptomic and proteomic expression differences based on HPV status included p16 (CDKN2A), RRM2, and CDC25C. These models will allow for the direct testing of targeted therapies in PSCC and determine their response in correlation to HPV status.

## 1. Introduction

For patients with metastatic penile squamous cell carcinoma (PSCC), the median survival time is <12 months, which decreases to less than 6 months after the disease becomes refractory to first-line therapy [1]. The overall response rate to common first-line combination chemotherapy treatments such as paclitaxel, ifosfamide, and cisplatin is 50% [2]. There is an urgent need in advanced-PSCC treatment to find novel therapies that would generate better response rates than standard empiric chemotherapy treatments and have less toxicity. Patient-derived xenograft models allow for direct testing and for mechanistic studies of targeted therapies, thus opening the door to new avenues of treatment.

Human papillomavirus (HPV) infection occurs in approximately 30 to 50% of PSCC cases [3]. There are >150 HPV subtypes; however, only high-risk HPV (HR-HPV) subtypes have been shown to be associated with PSCC, with HPV16 being the most prevalent associated genotype. HR-HPV is involved in the carcinogenesis of PSCC through the activity of viral E6 and E7 oncoproteins that bind to and inactivate p53 and retinoblastoma-1 tumor suppressor protein (Rb), respectively [4]. E7’s inhibition of the Rb pathway leads to increased expression of the p16^INK4a^ (CDKN2A) protein, (also known as p16). Overexpression of p16 can be detected by immunohistochemical (IHC) staining and has been found to be a reliable marker for HR-HPV infection in oropharyngeal SCC and PSCC [5,6,7,8,9]. PSCC patients with strong p16 expression observed via IHC have been found to have a better prognosis than PSCC patients with spotty expression or no expression [10,11]. Therefore, there is evidence that carcinogenesis is different in HPV-positive (HPV+) versus HPV-negative (HPV−) PSCC, and this could possibly be a therapeutic vulnerability. However, there are currently no approved targeted therapies for PSCC. One obstacle is the rarity of PSCC, which is an impediment to the clinical development of targeted therapies. Herein we report on our ability to generate and characterize seven patient-derived-tumor-xenograft (PDX) PSCC models using multiple-omics techniques toward the further development of preclinical testing of molecular strategies in penile cancer.

## 2. Materials and Methods

### 2.1. Patient Tissue

All patient tissues were obtained with written consent under research protocol PA16-0796 that was reviewed and approved by the MD Anderson Institutional Review Board. After surgical resection, patient tumor tissue was brought from the operating room to MD Anderson pathology, and any tumor tissue not needed for diagnosis or margin determination was placed in ice cold DMEM (90%, 10-013-CV, Corning, Corning, NY, USA) media with fetal bovine serum (FBS, 10%, F0926, MilliporeSigma, Burlington, MA, USA). This was then brought to the laboratory for processing. The whole process from the tissue leaving the operating room to the placement of the tissue in the animal varied but was approximately 4 h for most PDX attempts. Formalin-fixed, paraffin-embedded (FFPE) blocks of patient tumor tissue were used for hematoxylin and eosin (H&E) and p16 staining. In addition, unstained slides of patient tumor tissue (Pe821) and normal penile tissue (Pe821, Pe3, Pe9, Pe10, and Pe16) were used for DNA extraction. Clinical information for all patients was obtained through chart review.

### 2.2. Engraftment into Mice

Fresh tumor tissue was washed with phosphate buffered saline (PBS, 21-040-CV, Corning) containing 5 to 10% penicillin streptomycin (30-002 CL, Corning) and then the tissue was cut into 3 to 5 mm pieces. These pieces of tissue were subsequently placed in a 1:1 mixture of Matrigel (354262, Corning) with PBS on ice prior to engraftment. All animal work was approved and performed on ACUF protocol 1841 that was reviewed by the MD Anderson Institutional Animal Care and Use Committee. Male mice between the ages of 5 to 10 weeks were used for all engraftments. Either NSG (strain 00557 from Jackson Laboratory, Bar Harbor, ME, USA) or NCG (strain 572, Charles River, Houston, TX, USA) mice were used for the engraftment of tissues. A similar engraftment protocol to Palanisamy et al. was used [12]. For each engraftment, one piece of tissue approximately 3 mm in size was placed subcutaneously in the flank on both the right and left side of the mouse.

### 2.3. Tumor Growth Measurements

We determined the tumor-growth curves, time to tumor formation (TTF), and time to harvest (TTH) in 6 of our 7 PSCC PDX models, with 2 to 4 tumors per model throughout 3 generations or 3 passages. After initial engraftment from patient-resected tumor tissue, mice were monitored for tumor growth, and if growth did occur then this was considered passage 1 (P1). When tumors reached approximately 1500 mm^3^, mice were euthanized and tumor tissue was resected and treated in a similar manner to the original patient tissue. Tumors were harvested immediately after euthanasia and washed with phosphate buffered saline (PBS, 21-040-CV, Corning) containing 5 to 10% penicillin streptomycin (30-002 CL, Corning). Tumors were immediately cut into small pieces (~3 mm in size) and placed in 1:1 to Matrigel (354262, Corning) and PBS on ice. These pieces of tissue were then engrafted into 5 mice (2 pieces of tissue per mouse placed subcutaneously in the flank). The remaining tissue was either cryofrozen in liquid nitrogen, used for FFPE blocks, or placed in a mixture of 50% FBS (fetal bovine serum, F0926, MilliporeSigma), 40% DMEM (10-013-V, Corning), and 10% DMSO (D2650, MilliporeSigma) and frozen in a Nalgene Mr. Frosty (catalog 5100-001, ThermoFisher Scientific, Waltham, MA, USA) for future animation. Tumor growth was monitored weekly using calipers to measure the tumor’s length (L) and width (W). Tumor volume was calculated using the formula V = (L × W^2^) × 0.5. The mice were monitored daily for signs of morbidity and were sacrificed when the tumors reached a size of ~1500 mm^3^ or if they showed signs of distress. TTF was defined as the time in days to the first palpable tumor. TTH was defined as the time in months to collection of the tumor after reaching maximum tumor volume (~1500 mm^3^). Tumor growth curves, TTF, and TTH were plotted and analyzed using GraphPad Prism 9.00 software (Boston, MA, USA).

### 2.4. Statistical Analysis of Clinical Characteristics and Engraftment

The association of patient clinicopathologic characteristics and positive engraftment for P1, P3, and P4/5 for all tissues was determined. The clinical characteristics were compared using Wilcoxon Rank Sum test and Fisher’s exact test. R version 4.0.3 was utilized for analysis.

### 2.5. Histology and Immunohistochemical Staining (IHC)

Tissues were fixed in 10% neutral buffered formalin and processed routinely prior to paraffin embedding. Sections were cut at 4 μm thickness and stained with hematoxylin and eosin (H&E). For all patient tissues, histological subtype, tumor grade, perineural invasion (PNI), lymphovascular invasion (LVI), and p16 staining patterns were confirmed by a genitourinary pathologist (PR). Tumors from PDX models were analyzed by a veterinary pathologist (NF). Immunohistochemistry was performed on all original patient tumors and on PDX tissues for models XPe821, XPe3, XPe9, XPe10, XPe13, XPe16, and XPe20. For p16 IHC, all samples were tested and stained on a BenchMark Autostainer (Ventana Medical Systems, Tucson, AZ, USA) as described by the manufacturer’s protocol using a prediluted mouse monoclonal antibody (CINtec^®^ p16 Histology, clone E6H4, Ventana Medical Systems, Tucson, AZ, USA). Histology slides were digitally scanned by an Aperio AT2 whole slide digital scanner (Leica Biosystems, Vista, CA, USA) at 20× resolution and viewed with Image Scope v.12.4.6.

For p16 IHC analysis, staining patterns were classified as 0, 1, 2, or 3 using previously described categories [13,14]. We used our recently published hybrid system (HS) for analyzing p16 via IHC. The HS method uses both the percentage (>75%) of positive staining of the tumor section and the staining pattern [10]. In the HS method, tumor tissues with a positive staining of >75% and a pattern of 3 or 2 are considered positive for p16, whereas those with a staining pattern of <75% and a staining pattern of 2, 1, or 0 are considered negative for p16.

### 2.6. DNA Extraction

In most cases, DNA was extracted from cryofrozen PDX and patient tumor tissue using QIAmp DNA Mini Kits (51304, QIAGEN, Hilden, Germany) and then assayed using a NanoQuant Plate (TECAN, Mannedorf, Switzerland) or Qubit (Thermo Fisher Scientific). DNA was extracted from unstained FFPE histology slides for patient tumor tissue Pe821 and from normal penile tissue from patients Pe821, Pe3, Pe9, Pe10, and Pe16. For histology slides, DNA was extracted using the Ionic FFPE to Pure DNA Kit with the Ionic Purification System (Purigen Biosystems, Pleasanton, CA, USA), which enables the automated purification of DNA from FFPE tissue samples. Tumor and normal tissue were initially determined from all FFPE blocks via H&E and pathological review (PR). Microdissection was performed on samples containing both normal and tumor tissue for precise analysis.

### 2.7. STR Fingerprinting

Short tandem repeat (STR) DNA profiling was performed to authenticate the genomic DNA of PDX tumor tissue according to the original patient tumor tissue’s genomic DNA. STR fingerprinting relies on screening multiple regions of microsatellite instability in the genome. Fifty microliters of DNA at a concentration of around 30 ng/μL was assayed using the Promega Powerplex 16 HS kit (DC2100, Promega, Madison, WI, USA) that screens separate loci using PCR. All STR profiles were matched to public and in-house profiles. All STR fingerprints for penile patient tissue and PDX tumor tissue were found to be unique to the public and in-house profiles but were considered matches to each other.

### 2.8. Whole-Exome Sequencing (WES) and Analysis

A total of 20 unique samples were sequenced with a NovaSeq600 system (Illumina, San Diego, CA, USA) using 150-base pair end sequencing by Illumina with UMI. We aligned short reads against the hg38 human reference genome assembly using BWA (v0.7.17) [15]. For PDX samples, we used a human (UCSC hg38) + mouse (UCSC GRCm39) hybrid reference genome to remove mouse genome contamination [16]. We used matched normal tissue as a control to call somatic variations using VarScan (v2.4.2) [17]. In patient samples, DNA from patient tumor and normal tissue samples from Pe3, Pe9, Pe10, Pe16, Pe20, and Pe821 were utilized. In addition, we analyzed patient tumor DNA from Pe13 but, as it did not have a matched normal control, Pe20 normal tissue was used as a control. DNA from PDX tumor tissues XPe3 (P3), XPe9 (P3), XPe10 (P3), XPe13 (P3), XPe16 (P2), XPe20 (P1), and XPe821 (P2) were sequenced; passage numbers are in parentheses. We performed the annotation of somatic variants using annoVar [18]. Depth of coverage for sequencing was on average 176 + 50x coverage. For patient Pe821, due to poor quality DNA from the FFPE tumor block, variants with strand bias *p* < 0.05 by Fisher’s exact test were removed. We also demanded that the alternative alleles have 2 or more reads support for each strand (alt_plus ≥ 2 reads, alt_minus ≥ 2 reads). We focused on determining mutations in tier 1 genes from the cancer gene census database. Tier 1 genes most possess two things—(1) documented activity relevant to cancer and (2) evidence that mutations within the gene changes activity of the protein promoting oncogenic transformation [19].

### 2.9. RNA Extraction and Sequencing

Total RNA was extracted from cryofrozen PDX tissue and original patient tissue using the RNeasy Mini Kit (74106, QIAGEN) and it was then assayed using a NanoQuant Plate or Qubit. A total of 14 RNA samples were sequenced using Illumina NextSeq500 (7 patient-tumor tissue samples (Pe9, Pe10, Pe13, Pe16, Pe18, Pe20, and Pe25) and 6 PDX samples (XPe3 (P3), XPe9 (P3), XPe10 (P3), XPe13 (P3), XPe16 (P2), and XPe821 (P2)); passage numbers are in parentheses.

To remove the mouse-transcript contamination in the RNA-Seq data, we used a hybrid human–mouse genome reference (UCSC human genome GRCh38 + mouse genome GRCm39) for all samples. We used STAR Aligner [20] for RNA-Seq alignment and quantified known human transcripts using the Gencode database (v31). To assess the expression levels of genes in the HPV16 genome, we added HPV16 gene structures from the PaVE database ([21], https://pave.niaid.nih.gov) to the Gencode dataset and quantified them with the transcript quantification tool Salmon [22]. To perform differential gene expression analysis, we used limma [23] and edgeR [24] packages. Batch correction between human tissue and PDX tissue was performed using Bioconductor package sva’s ComBat function [25]. The gene set enrichment analysis was performed on differentially expressed genes using Bioconductor package topGO. To determine the immune cell decomposition from bulk RNA-Seq data, we used a mouse MCP counter [26]. MCP counter is a computational method that quantifies the immune and non-immune stromal cells in a tissue sample based on the transcriptome.

### 2.10. Reverse Phase Protein Array (RPPA)

Cryofrozen tissue from 3 to 4 separate tumors (biological replicates) from 6 different PDX models (XPe3, XPe9, XPe10, XPe13, XPe16, and XPe821) were given to the MD Anderson reverse phase protein array core (https://www.mdanderson.org/research/research-resources/core-facilities/functional-proteomics-rppa-core.html) for processing. The tissues were treated in a similar manner to published procedures [27,28,29]. Approximately 400 proteins are assayed using RPPA. A second RPPA analysis was performed on frozen patient tumor tissue from Pe3, Pe9, Pe10, Pe13, Pe14, Pe16, and Pe18.

For PDX tissue RPPA, a linear mixed model (LMM) was used to assess the differences in protein expression between HPV-positive and HPV-negative PDX samples on a feature-by-feature basis. The least-squares means by HPV status were also estimated. For the patient tissue RPPA, the two sample t-test was used to assess the differences in protein expression between HPV-positive and HPV-negative patient tissue on a feature-by-feature basis. For both analyses, the linear FC (fold change) values were calculated as the estimated ratio between the 2 groups under comparison, with the following conventional modification—for the ratios > 1 (up-regulation), FCs were noted as the same as the ratio. For the ratios < 1 (down-regulation), FCs were noted as the negative inverse of the ratio. To account for multiple testing, we estimated the false discovery rate (FDR) of the overall test of the model using the Benjamini–Hochberg method. For both analyses, R version 4.0.3 was utilized.

### 2.11. Immunoblots

Cryofrozen PDX tumor tissue was ground into a fine powder using a liquid nitrogen-cooled stainless steel mortar and pestle. This cryofrozen powder was then added to a 15 mL falcon tube with approximately 3 mL of RIPA buffer (89900, Pierce RIPA Buffer, Thermo Fisher Scientific, Waltman, MA, USA) with 1x phosphatase and protease inhibitor (Halt Protease and Phosphatase Inhibitor Cocktail, 1861281, Thermo Fisher Scientific). This was further homogenized on ice with a Polytron PT2500C with a PT-DA 03 wand (Kinematica, Bohemia, NY, USA). Lysates were centrifuged for 15 min at 4 °C at 14,000 g and the supernatant removed. Protein concentration was determined using a BCA assay (DC Protein Assay, 5000112, Bio-Rad, Hercules, CA, USA). A total of 20 μg of protein for each sample was loaded on 4–20% Mini-PROTEAN pre-cast TGX gels (4561094, Bio-Rad,) and the gels were then transferred to PVDF membranes (1620174, Bio-Rad). Membranes were blotted with primary antibodies for RRM2 (1:1000, 65939S, Cell Signaling, Danvers, MA, USA); p16 (1:1000, 80772S, Cell Signaling); CDC25C (1:1000, 4688S, Cell Signaling); and HPV16-E7 (1:1000, GTX133411, GeneTex, Irvine, CA, USA) and a corresponding secondary antibody (1:2000 of Anti-rabbit IgG HRP linked, 7074P2, Cell Signaling or 1:2000 Anti-mouse IgG HRP linked, 7076S, Cell Signaling). All membranes were imaged using chemiluminescent HRP substrate (WBKLS0500, MilliporeSigma) on an Azure 300 instrument (Azure Biosystems, Dublin, CA, USA). On one immunoblot, lysate from the head and neck HPV− cell line HN31 was utilized as a negative control.

## 3. Results

### 3.1. Tumor Engraftment Rate and Growth Rate

We attempted to generate PDX models from 19 tumor tissues from 18 consented patients (2 tissues came from the same patient). We had a success rate of 50% for the initial passage (P1) as defined as the direct engraftment of patient tissue into immunocompromised mice. Tumors that engrafted were then passaged in a new subset of mice (P2, P3, P4, and P5). For initial engraftment attempts (P1), tissue was placed in the mice prior to analysis of the clinical pathology report. In three cases (XPe7, XPe12, and XPe17), we attempted to engraft excised patient tissue in which, based on pathology, no tumor was present. Clinical variables associated with each tumor tissue are given in Table S1, and the *p*-values for clinical characteristics associated with successful engraftment are given in Table S2.

We characterized seven models from separate patients that were passaged to P4 or greater. These models included four HPV+ models (XPe821, XPe3, XPe9, XPe16) and three HPV− models (XPe10, XPe13, XPe20). Table 1 summarizes the clinical data and Figure S1 is a graphical depiction of the treatment that each patient from our seven PDX models received. We analyzed tumor growth curves in XPe821, XPe3, XPe9, XPe10, XPe13, and XPe16 PSCC models (Figure S2).

### 3.2. STR Characterization and HPV Genotyping

Short tandem repeat (STR) fingerprinting for the donor tissue (patient), P1, and P4/5 of our seven models are shown in Table 2. STR fingerprints for the remaining P1 models are shown in Table S3. We observed genomic instability between donor, P1, and P4 tissues in the XPe20 model.

Using RNA isolated from patient tumor tissue and PDX tissue, transcript counts for HPV16-E6, HPV16-E6* (shortened version of the original transcript), and HPV16-E7 were determined [21]. High levels of HPV16-E6, HPV16-E6*, and HPV16-E7 were observed in patient tissue for Pe9 and Pe16 and in PDX tissue for XPe821, XPe3, XPe9, and XPe16 (Table 3).

### 3.3. Histology

**Figure 1:**
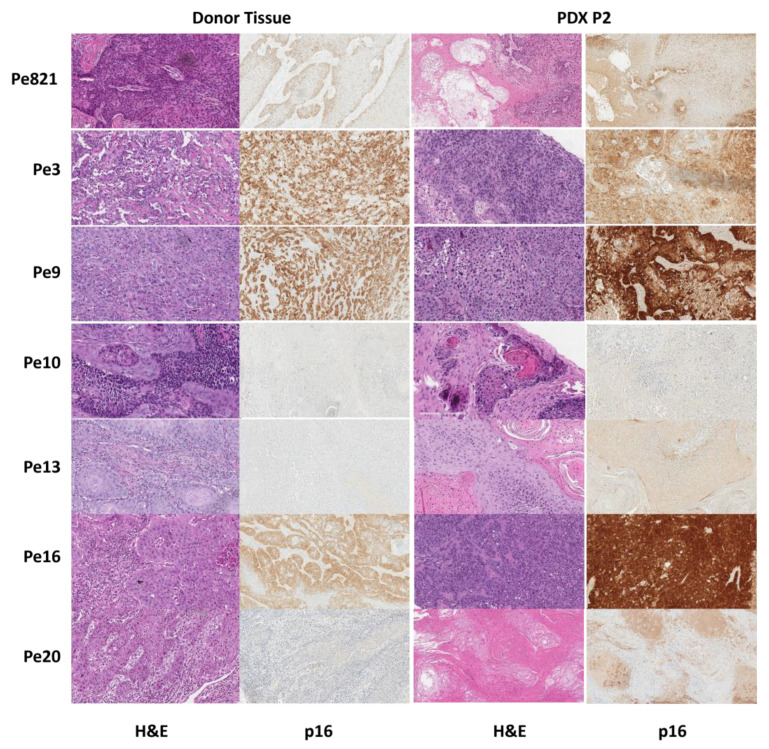
H&E and p16 IHC images for original patient tissue (donor) and passage 2 of the PDX model (P2).

Hematoxylin and eosin (H&E) and p16 staining were performed on both the donor and the PDX tissues. The H&E and p16 staining performed on patient tissue and P2 of PDX tissues for all seven models are shown in Figure 1. The p16 staining pattern [13,14] and the percentage of positive staining were determined for all patient-donor tumor tissues and for the P2 of each PDX model. Using our hybrid method, XPe3, XPe9, and XPe16 were p16 positive, while XPe8, XPe10, XPe13, XPe20, and XPe821 were p16 negative. Both XPe821 and XPe20 had a staining pattern of one and <75% staining.

### 3.4. Proteomics

**Figure 2:**
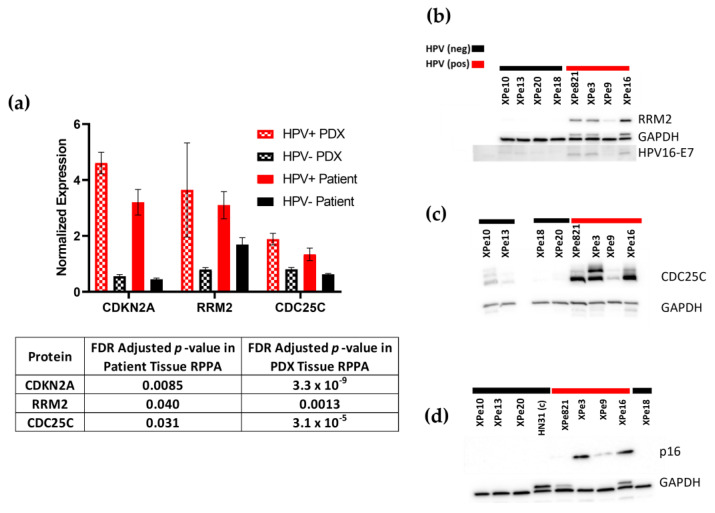
Differentially expressed proteins based on HPV status. (**a**) Normalized linear expression of RRPA data of CDKN2A, RRM2, and CDC25C depicted in a bar blot with false discovery rate (FDR) adjusted *p*-value given for each protein per data set. (**b**) Immunoblot of lysates for HPV+ (red bars) and HPV− (black bars) PDX models for RRM2, HPV16-E7, and GAPDH depicted. (**c**) Immunoblot of CDC25C and GAPDH. (**d**) Immunoblot of p16 and GAPDH. The uncropped blots are shown in File S1.

Cryofrozen tumor tissues from PDX models (XPe3, XPe9, XPe10, XPe13, XPe16, and XPe821) were assayed by reverse-phase protein array (RPPA) analysis. RPPA analysis determines the expression of ~400 proteins with absolute quantification of protein expression and modification. Another RPPA analysis was performed using cryofrozen patient tumor tissues (Pe9, Pe10, Pe13, Pe14, Pe16, and Pe18). Differential protein expression based on HPV status was determined in both patient and PDX tumor samples, and eighteen proteins were found to be selectively expressed (Table S4). Large differences in expression based on HPV status that were found by RPPA analysis occurred in proteins CDKN2A, RRM2, and CDC25C (Figure 2a), and this was confirmed by immunoblotting (Figure 2b–d).

### 3.5. Transcriptomics

**Figure 3:**
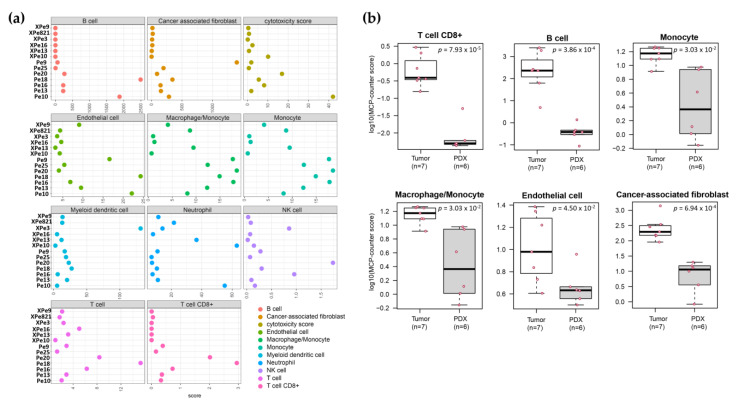
RNA-Seq Data Analysis. (**a**) MCP-counter deconvolution revealed differences in the population of microenvironment cells such as B cells, cancer-associated fibroblast, monocytes, macrophages, and activated CD8+ T cells in patient tumor tissues labeled Pe9, Pe10, Pe13, Pe16, Pe18, Pe20, and Pe25 compared to PDX tissues labeled XPe3, XPe9, XPe10, XPe13, XPe16, and XPe821. (**b**) Box plots of different cell populations found to be highly expressed in patient tumor samples compared to PDX tissues. *p*-values were obtained from t-test with FDR adjustment.

RNA-Seq was performed on total RNA isolated from 13 samples (7 patient and 6 PDX samples). The number of RNA pairs sequenced for each sample is given in Figure S3. After removing mouse-transcript contamination, 1783 genes were found to be differentially expressed between PDX tissues and their corresponding patient tumor tissue (Pe9, Pe10, Pe13, and Pe16; Figure S4). The majority of differentially expressed genes in patient versus PDX tissue are involved in immune-related pathways (Table S5). This differential expression of immune genes was further analyzed using the microenvironment cell populations-counter (MCP-counter) method to determine the abundance of immune and stromal cell populations in the tissues [26]. We observed a significantly higher population of CD8+ T cells, B cells, monocytes, macrophage/monocytes, endothelial cells, and cancer-associated fibroblasts in the patient tumor samples compared to the PDX samples (Figure 3).

To get a better understanding of the variability in transcription levels due to HPV status, we performed batch correction between primary tumors and PDX samples using Surrogate Variable Analysis (SVA) [25]. We found that 590 genes were differentially expressed based on HPV status, and the top 19 biological processes associated with these gene differences were determined using GO ontology terms and gene set enrichment analysis (Table S6) [30]. In addition, we determined the transcript levels for the 18 genes that matched the proteins found by RPPA to be differentially expressed based on HPV status. We observed the transcript levels of CDKN2A, MSH6, RRM2, and CDC25C to be significantly higher in HPV+ versus HPV− tissues (FDR Adjusted *p* < 0.05).

### 3.6. Genomics

**Figure 4:**
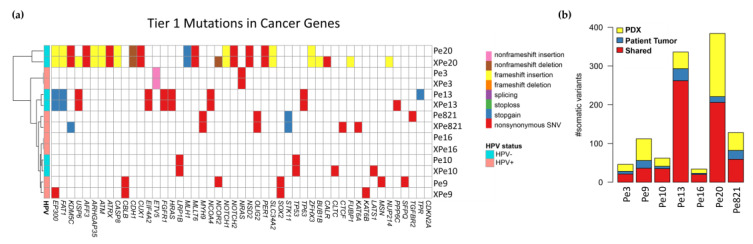
(**a**) Mutations in tier 1 genes from the cancer gene census database. (**b**) Somatic variants found only in the PDX tissue (yellow), only in the patient tumor tissue (blue), or shared between the patient tumor tissue and the PDX tissue (red).

Whole-exome sequencing (WES) was performed on a total of 20 unique samples. DNA from both patient tumor and matched normal tissue samples for Pe3, Pe9, Pe10, Pe16, Pe20, and Pe821 were utilized. We analyzed patient tumor DNA for Pe13, but as it did not have a matched normal control Pe20 normal tissue was used as a control. DNA for PDX tumor tissues XPe3, XPe9, XPe10, XPe13, XPe16, XPe20, and XPe821 were sequenced. The number of somatic variants with coding-change consequences is given in Table S7. A heatmap showing the mutations in tier 1 genes from the cancer gene census database found in tumor tissues and PDX tissues is illustrated in Figure 4a. Most mutations present in the patient tumor tissue were conserved in PDX tissue (Figure 4b, Table S8). We observed a higher number of mutations for all PDX-tissue models versus the donor tissue. The percentage of shared somatic mutations in donor and PDX tissues when compared to the total number of mutations found in the PDX tissue was 59.7 + 14.2% (average + standard deviation); however, this percentage ranged from 85.9% (Pe13) to as low as 39.1% (Pe9). We did not observe a grouping of genetic mutations based on HPV status.

### 3.7. APOBEC Mutations

**Figure 5:**
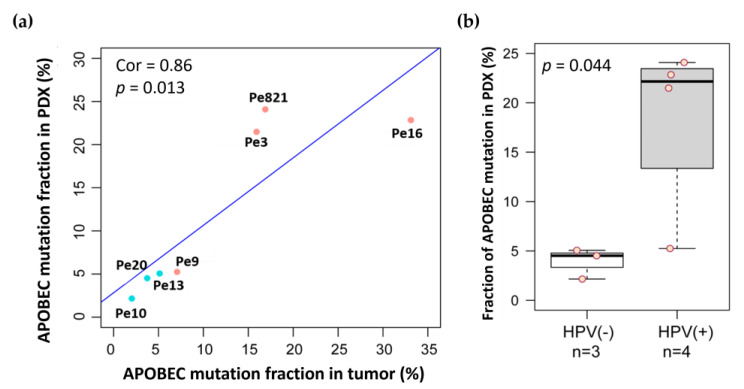
(**a**) APOBEC mutation (C-to-T and C-to-G mutation in TCW context) fraction percentage in original patient tumor (*x*-axis) and PDX tissue (*y*-axis) plotted. Pink circles are HPV+ tissues while blue circles are HPV− tissues. (**b**) The APOBEC mutation fraction in the PDX tissues alone are shown in the box and whiskers plot.

Apolipoprotein B mRNA editing catalytic polypeptide like (APOBEC) proteins are associated with protecting mammalian cells from viral infections [31]. APOBEC activity leads to a higher number of C-to-T and C-to-G mutations in a TCW trinucleotide context; therefore, we determined the fraction of C-to-T and C-to-G mutation in a TCW context from both the original patient tumor and the PDX tissue of our models (Figure 5). We did observe a higher fraction of APOBEC mutations in HPV+ tissues (XPe821, XPe3, and XPe16). We also observed similar APOBEC mutational fractions in both donor tumor tissue and the PDX tissue (Pearson correlation coefficient r = 0.86). The APOBEC mutational fraction was also higher in the HPV+ versus HPV− PDX tissue (*p* = 0.044).

## 4. Discussion

We generated seven PDX models of PSCC that were found to represent the original patient tumor tissue in STR, p16, and histology, which is similar to other PSCC PDX models generated in other laboratories [32,33,34]. Using MCP-counter deconvolution, an increased population of microenvironment cells such CD8+ T cells, B cells, monocytes, macrophages, endothelial cells, and cancer-associated fibroblast cells were found in the donor tissue compared to the PDX tissue. This correlates with what has been found in other PDX models [35], where only the cancer cells are passaged in the model and not human immune cells. This could potentially be abrogated to some extent, however, by using humanized NSG mice when generating PDX models [36], and this is an area of future growth for PSCC research.

To further explore the differences in transcript expression due to HPV status, we batch corrected the two data sets in Bioconductor [25] and differential expression analysis was performed. Due to our small sample number, we considered the PDX samples to be independent of the corresponding patient tumor sample. Using each tissue as an independent variable, we had seven HPV− and six HPV+ tissues in the analysis. Utilizing GO pathway analysis, we found differential expression based on HPV status in multiple pathways, including DNA replication, meiotic cell cycle, cell division, and regulation of cell cycle pathways. This correlates with what has been found in head and neck SCC [37,38] and with the high expression of CDKN2A found in our HPV+ tissues. CDKN2A or p16 is a cell cycle-regulation protein that is considered to be a tumor suppressor [39], but in HPV+ malignant tissue it is upregulated [36]. In addition, we observed the transcript levels of CDKN2A, RRM2, and CDC25C to be higher in HPV+ tissues versus HPV− tissues (FDR Adjusted *p* < 0.05). The increased mRNA levels of these proteins correlated with the higher protein expression observed by RPPA analysis.

Based on the transcriptomic and RPPA data, we validated the expression of CDKN2A (p16), CDC25C, and RRM2 in our PDX models. For p16, IHC was used to determine expression in both patient donor and PDX tissues. Positive p16 expression was found in XPe3, XPe9, and XPe16 lysates in the immunoblot that correlated with the p16-positive assignments using our hybrid IHC-scoring method. For XPe20 and XPe821, PDX lysates had little to no p16 expression based on immunoblotting and they were considered p16-negative using our hybrid IHC-scoring method [10]. We did not observe HPV16 E6 or E7 transcripts in Pe20 patient tissue and we observed the transcripts for both proteins in XPe821. Therefore, we considered the XPe821 model to be HPV+ and the XPe20 model to be HPV−. Even with low p16 expression, model XPe821 has a higher APOBEC mutational burden and higher expression of RRM2 and CDC25C compared to HPV− tissues. We observed higher expression of RRM2 and CDC25C in HPV+ PDX lysates compared to HPV− PDX lysates. RRM2 (ribonucleoside-diphosphate reductase subunit M2) is utilized in the synthesis of deoxynucleoside triphosphate (dNTP) during the S phase of the cell cycle. RRM2 has been shown to be overexpressed in HPV+ cervical SCC [40] and it is associated with worse overall survival in oral SCC patients [41]. CDC25C (cell division cycle 25C) dephosphorylates cyclin B-bound CDC2 and initiates entry into mitosis [42]. Its overexpression has been found to be associated with malignant features and aggressive cancer phenotypes in vulvar SCC and cervical SCC [42].

We performed non-targeted WES on original patient tumor tissue and PDX tissues. The tier 1 cancer gene mutations found in the patient tissue in all models but Pe9 are found in the PDX tumor tissue. For Pe9, only two of the five tier 1 mutations were found in the PDX genome. For all models, more mutations were found in the PDX tissues than in the parental tumor tissue. This is similar to the higher number of mutations per megabase found in colorectal xenograft models (14.7) versus the parental tumors (10.9) [43]. Variability in the number of single nucleotide variances (SNVs) between PDX models and original tumor tissue has also been associated with clinical features. In pediatric T-cell leukemia, relapsed cancer PDX models had more variable SNVs than PDX models generated from relapse-free patient tissue [44]. This could explain why we see little variability in the SNVs in our Pe16 model derived from a patient with a successfully treated primary tumor who is without metastases or recurrence. However, the Pe20 model’s patient tissue and PDX tissue had higher numbers of mutations compared to the other tissues. We hypothesize this could be because of its MLH1 mutation. MLH1 codes for a protein in the DNA mismatch repair pathway and, mutations in MLH1 generate microsatellite instability and elevate the spontaneous mutation rate during replication [45]. Genomic instability was observed in the STR fingerprinting of the XPe20 model. Interestingly, this patient developed bone metastases, which is rare in PSCC. We observed higher C-to-T and C-to-G mutations in our HPV+ samples compared to our HPV− samples. These mutations are thought to occur in HPV-associated cancers by the activity of APOBEC proteins, which are switched on by viral infection [31]. We observed similar APOBEC mutational fractions in both donor tumor tissue and the PDX tissue (Pearson correlation coefficient r = 0.86). A similar phenomenon was observed in oral SCC, where much higher numbers for C-to-T and C-to-G mutations were observed in the 149 HPV+ oral SCC tissue samples versus the 335 HPV− oral SCC tissue samples [46]. We recently found that patients with PSCC enriched for APOBEC mutational patterns had higher tumor mutational burdens and exhibited worse overall survival than the non-APOBEC-enriched subset [47].

The first PSCC PDX was reported by Thomas et al. [32]. Recently, this same laboratory created 11 PSCC PDX models that were passaged three times. These models were derived from either local recurrence, lymph node resection, or a metastatic primary lesion [33]. We have similar engraftment rates (50%) for the first passage when compared with the 61% engraftment rate of Elst et al. [33]. Most of our models are derived from metastatic PSCC except for XPe16. XPe16 was generated from a primary PSCC tumor that did not receive neoadjuvant treatment prior to resection and the patient is currently disease-free two years after a partial penectomy. Another difference in comparing the present study to Elst et al. relates to genomic mutations found among tier 1 genomic alterations. We noted a single model (XPe10) with a TP53 mutation, one model with a NOTCH1 mutation (XPe20), and no models with CDKN2A mutations or TERT mutations. This differs from the Elst et al. study, where six models had mutations in TP53, four models had CDKN2A mutations, four models had TERT promoter mutations, and four had NOTCH1 mutations. These differences probably arise in part from the two different methods used for genome sequencing. We employed a non-targeted WES approach whereas Elst et al. utilized a targeted-sequencing approach focusing on protein-coding exons, promoter regions, and/or intronic regions of 96 cancer genes [33]. PDX models in the current study exhibited similar WES mutations to those observed in the non-targeted approach taken with 34 PSCC patients that was reported by Chahoud et al. [47].

There are currently two reported transgenic animal models of PSCC. The HPV16-positive model was generated by exposing K14HPV16 mice to DMBA (dimethylbenz[a]anthracene) [48]. K14HPV16 mice express HPV16 genes under the control of cytokeratin 14 gene promoter. The other transgenic mouse model is HPV-negative and was created using the co-deletion of Smad4 and Apc in the epithelium of the penis [49]. Using this model, immune checkpoint blockade (ICB) was found to reduce tumor growth and give enhanced tumor regression when combined with cabozantinib or celecoxib [49]. These models, along with PSCC PDX models, have opened the door to finding and testing targeted therapies for PSCC in a preclinical setting.

Our models have added to the understanding of PSCC but also reveal the limitations of subcutaneous PDX models such as the loss of the immune microenvironment of the original patient tissue, limitations in the evaluation of metastatic progression as the tissue is not placed orthotopically, and differences in exome-mutational burden and transcript levels between PDX tissues and original patient tissues. We plan to validate the differences observed in our study between HPV+ and HPV− PSCC in a larger cohort of tissues and PDX models.

## 5. Conclusions

We generated HR-HPV-positive and HR-HPV-negative PSCC PDX animal models that resembled human donor tissue at the histopathologic and molecular levels. We found significant transcriptomic, proteomic, and APOBEC-mutational-fraction differences based on HPV status in PSCC patient and PDX tissues. The models developed in this study will potentially help to unlock the heterogeneity of pathobiology in PSCC in addition to enabling the development of targeted therapies.

## Figures and Tables

**Table 1:** Patient and donor-tissue characteristics.

Model Name	Age	Ethnicity	Tumor Site	HR-HPV	Histology	Path. Staging	Recurrence	Neoadj. Treatment
XPe821	68	White	Penis	16	basaloid	rpT4NxM1	yes	yes
XPe3	67	White	Penis	16	basaloid	rpT2Nx	yes	
XPe9	33	Latino	Lymph Node	16	SCC	pT3N3Mx	yes	yes
XPe10	80	Latino	Penis		SCC	pT3N3Mx		yes
XPe13	67	Latino	Penis		SCC	pT3Nx		
XPe16	82	White	Penis	16	basaloid	pT3N0		
XPe20	72	Latino	Penis		SCC	pT3Nx		

**Table 2:** Short tandem repeat fingerprinting (STR) of original patient tissue (Donor) and the PDX tissue XPe for initial passage (P1) and passage 4 (P4) or passage 5 (P5) for each model.

Tumor Tissue	CSF1PO	D13S317	D16S539	D18S51	S21S11	S5S818	D7S820	D8S1179
Pe821 Donor	10, 12	11	9, 10	15, 16	28, 29	11, 13	12	13, 14
XPe821 P1	10, 12	11	9, 10	15, 16	28, 29	11, 13	12	13, 14
XPe821 P5	10, 12	11	9, 10	15, 16	28, 29	11, 13	12	13, 14
Pe3 Donor	12	9, 11	9	12, 13	27, 33.2	11, 13	10, 12	13, 16
XPe3 P1	12	9, 11	9	12, 13	27, 33.2	11, 13	10, 12	13, 16
XPe3 P5	12	9, 11	9	12, 13	27, 33.2	11, 13	10, 12	13, 16
Pe9 Donor	7, 10	11	12	12, 14	29	11, 12	12	13
XPe9 P1	7, 10	11	12	12, 14	29	11, 12	12	13
XPe9 P5	7, 10	11	12	12, 14	29	11, 12	12	13
Pe10 Donor	11, 12	12	11, 12	12, 14	31, 32.2	11, 13	10, 12	12, 13
XPe10 P1	11, 12	12	11, 12	12, 14	31, 32.2	11, 13	10, 12	12, 13
XPe10 P5	11	12	11, 12	12, 14	32.2	11	10, 12	12, 13
Pe13 Donor	10, 12	9	10, 12	12, 15	30, 32	12	10, 12	11, 14
XPe13 P1	10, 12	9	10, 12	12, 15	30, 32	12	10, 12	11, 14
XPe13 P4	10, 12	9	10, 12	12, 15	30, 32	12	10, 12	11, 14
Pe16 Donor	11	11, 14	12, 13	12, 16	28, 29	9, 11	9, 10	12, 14
XPe16 P1	11	11, 14	12, 13	12, 16	28, 29	9, 11	9, 10	12, 14
XPe16 P5	11	14	12, 13	12	28, 29	9, 11	9, 10	12, 14
Pe20 Donor	12	9, 13,14	11, 12	16, 17	29, 30	7, 11	8, 11, 12	13, 15
XPe20 P1	12	9, 14	11, 12	16	30	7, 11	8, 12	13, 15
XPe20 P4	11, 12	9, 15	11	16	30, 31	7, 11	8, 12	14, 15

**Table 3:** HPV-16 transcript-expression levels in TPM (transcripts per million read) values.

Tumor Tissues	HPV16-E6	HPV16-E6*	HPV16-E7
XPe3 P3	319	1335	1402
Pe9 Donor	127	1200	524
XPe9 P3	466	4049	2091
Pe16 Donor	379	1432	892
XPe16 P2	329	1426	899
XPe821 P2	377	3119	1367
Pe10 Donor	0	0	0
XPe10 P3	0	0	0
Pe13 Donor	0	0	0
XPe13 P3	0	0	0
Donor Pe20	0	0	0

## Data Availability

The data in this study were generated by the authors and are available upon request from the corresponding authors.

## Supplementary Materials

The following supporting information can be downloaded at https://www.mdpi.com/article/10.3390/cancers16051066/s1, Table S1. Clinical Variables associated with tumor tissue taken from 19 patients. Table S2. Statistical analysis of clinical characteristics of patients and tumors and successful engraftment in passage 1 (P1), passage 2 (P2), and passage 4/5 (P4/5). Figure S1. Graphical depiction of the clinical treatment patients received and what resected tissue was used to generate models XPe821, XPe3, XPe7, XPe9, XPe10, XPe13, XPe16, and XPe20. Figure S2. Variability observed in tumor growth curves, time to tumor formation (TTF), and time to tumor harvest (TTH) between models and between passages for six models. Table S3. STR fingerprinting comparing donor tissue to PDX passage 1 (P1) of models XPe7, XPe8, and XPe18. Table S4. The 18 proteins found to be differentially expressed based on HPV status in both RPPA data sets. Figure S3. Summary of RNA-Seq data. Figure S4. RNA-Seq analysis of differentially expressed genes in PDX tissue (labeled with XPe) and patient tissue (labeled Pe). Table S5. Top 20 GO pathways upregulated in patient tumor tissue versus PDX tissue by RNA-Seq analysis. Table S6. Gene set enrichment analysis using GO ontology terms for differentially expressed genes based on HPV status. Table S7. Number of somatic variants with coding-change consequences in both patient tumor samples (Pe) and in the PDX tissue samples (XPe). Table S8. Number of somatic variants found in donor tissue, in PDX tissue, and in both tissues; File S1: Raw Blots.
