# Supplementary material for: Transcriptomic, Proteomic, and Genomic Mutational Fraction Differences Based on HPV Status Observed in Patient-Derived Xenograft Models of Penile Squamous Cell Carcinoma
Source: Cancers (Basel). 2024 Mar 6;16(5):1066. doi: 10.3390/cancers16051066 (PMC10930474; doi:10.3390/cancers16051066)
Supplement: Supplementary file 1 [file cancers-16-01066-s001.zip › File S1.pdf]

Raw Blots

Supplemental material for reviewers

All MW numbers are given in kDa

Combined Bands Image

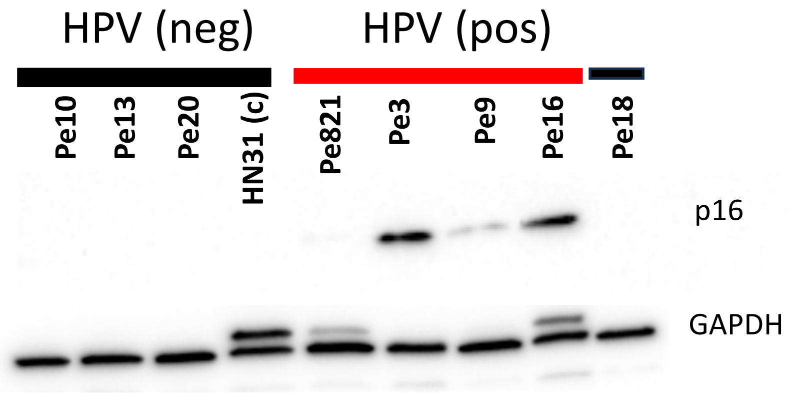

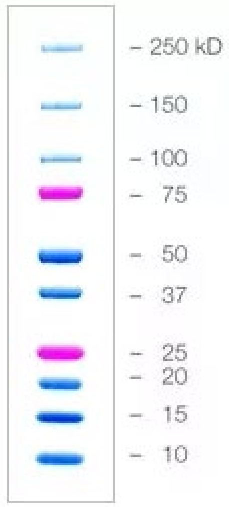

MW ladder used in all blots

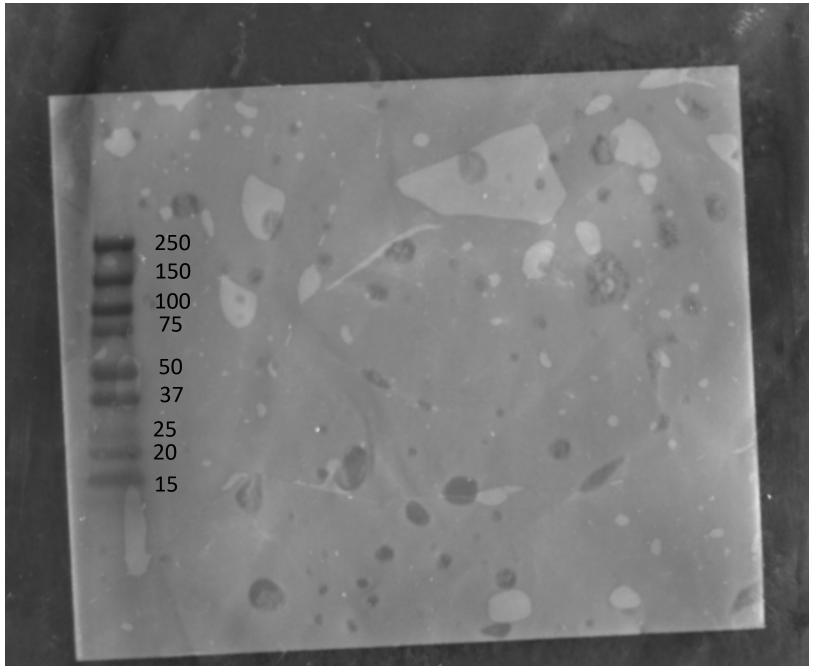

Bright field Image

Immunoblot p16

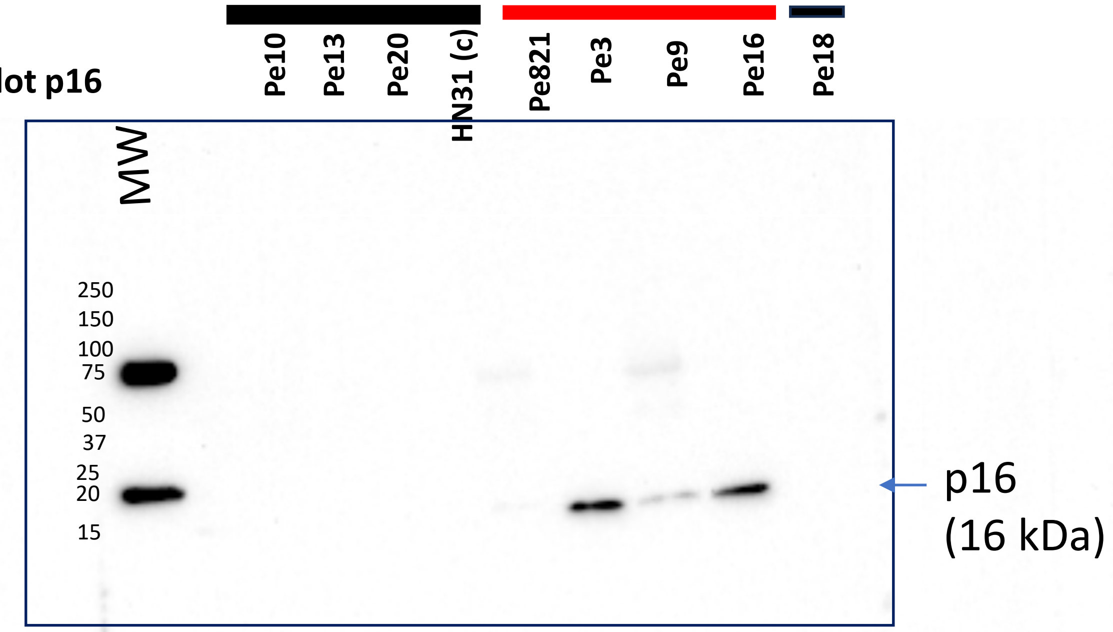

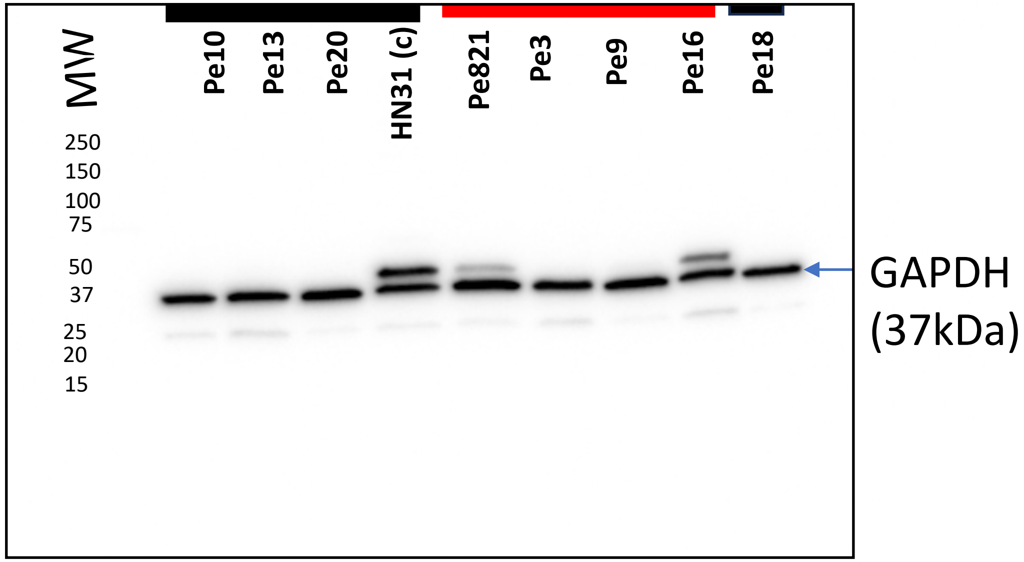

Immunoblot GAPDH

All MW numbers are given in kDa

Combined Bands Image

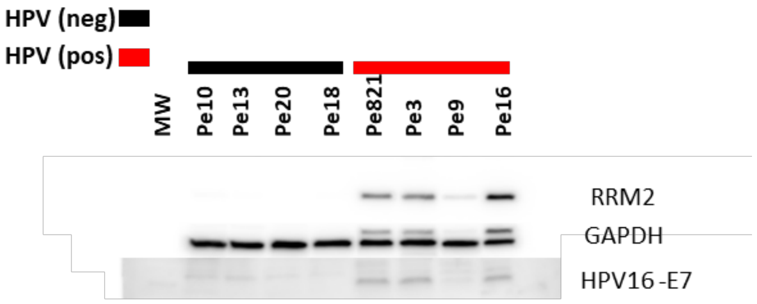

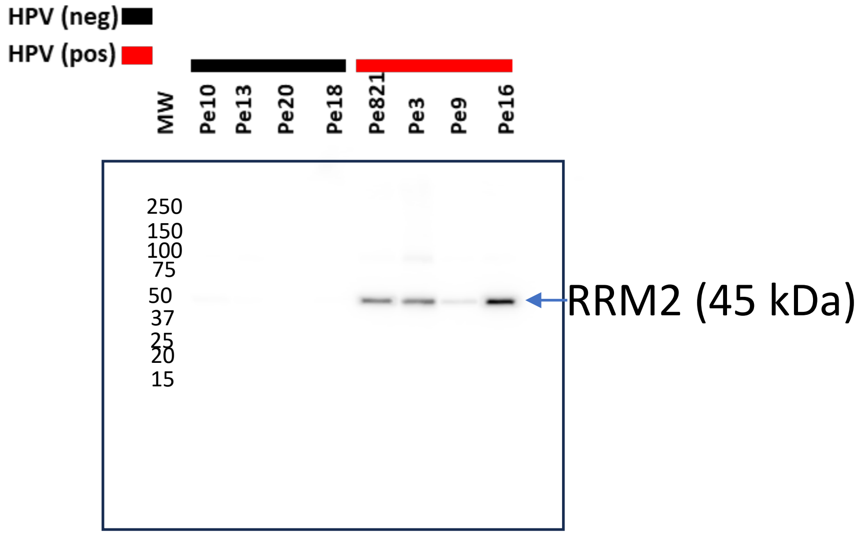

Immunoblot RRM2

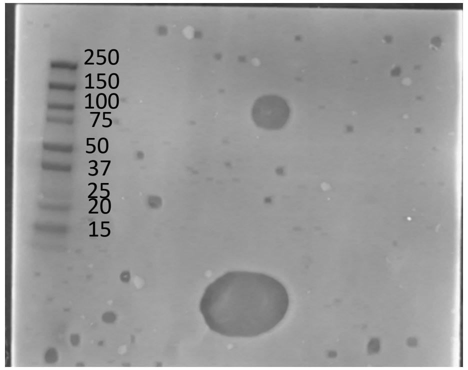

Bright field Image

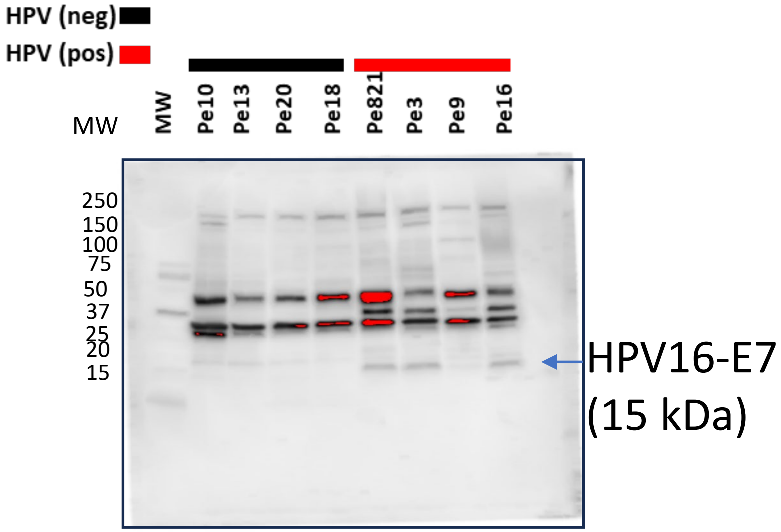

Immunoblot HPV16-E7

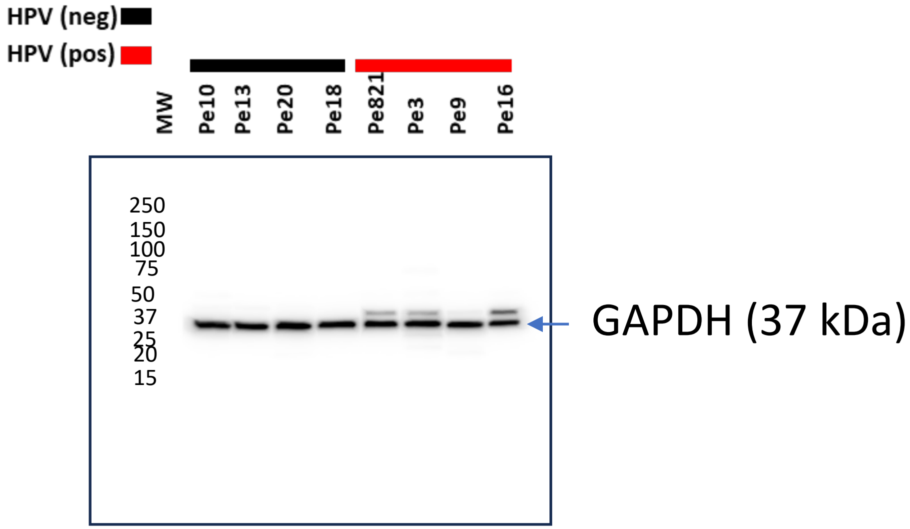

Immunoblot GAPDH

All MW numbers are given in kDa

HPV (neg)

HPV (pos)

Combined Bands Image

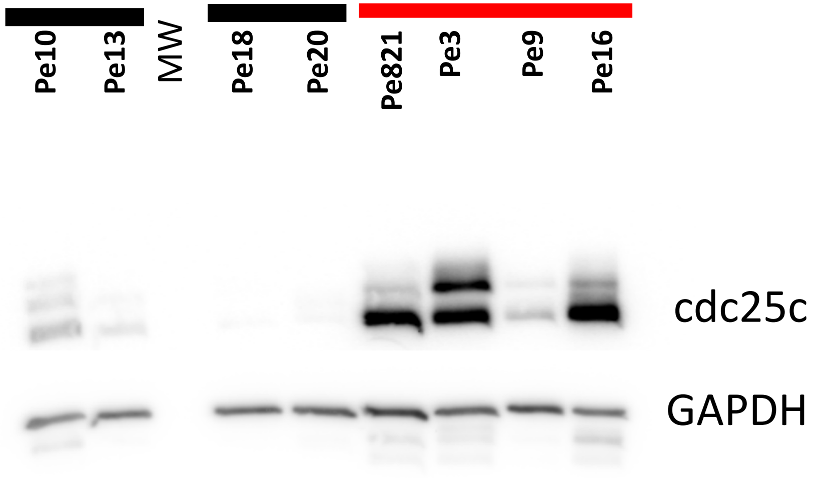

Bright field Image

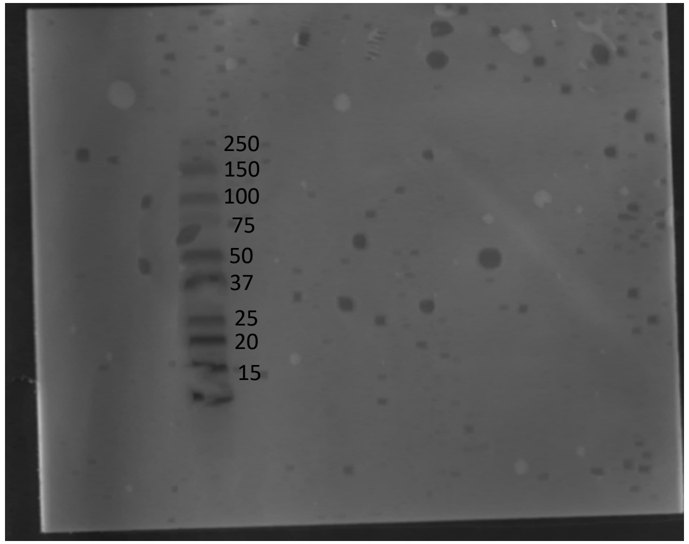

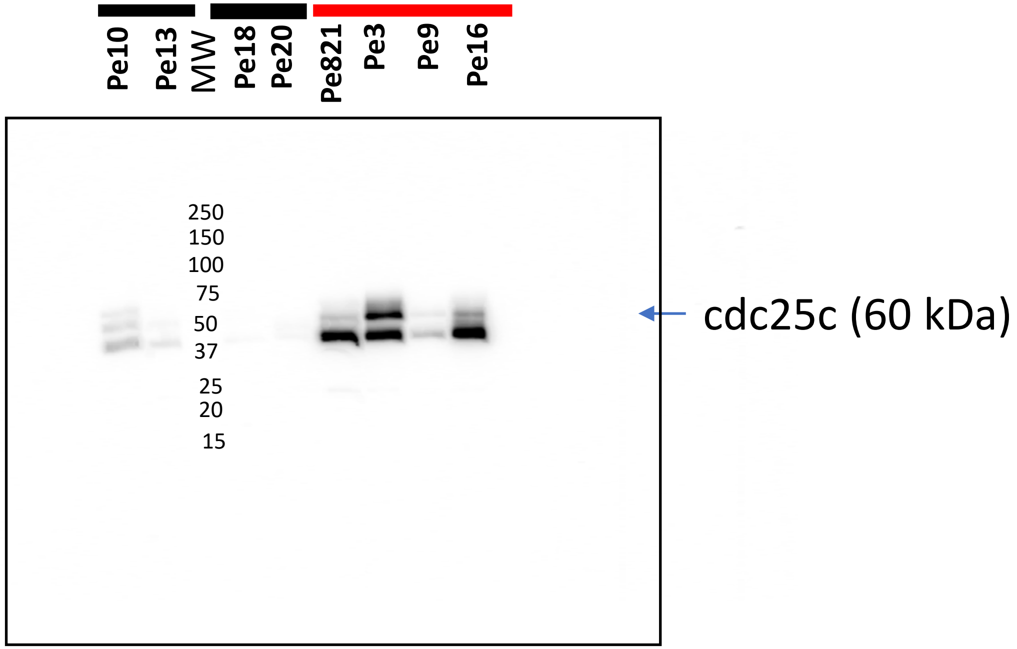

Immunoblot CDC25C

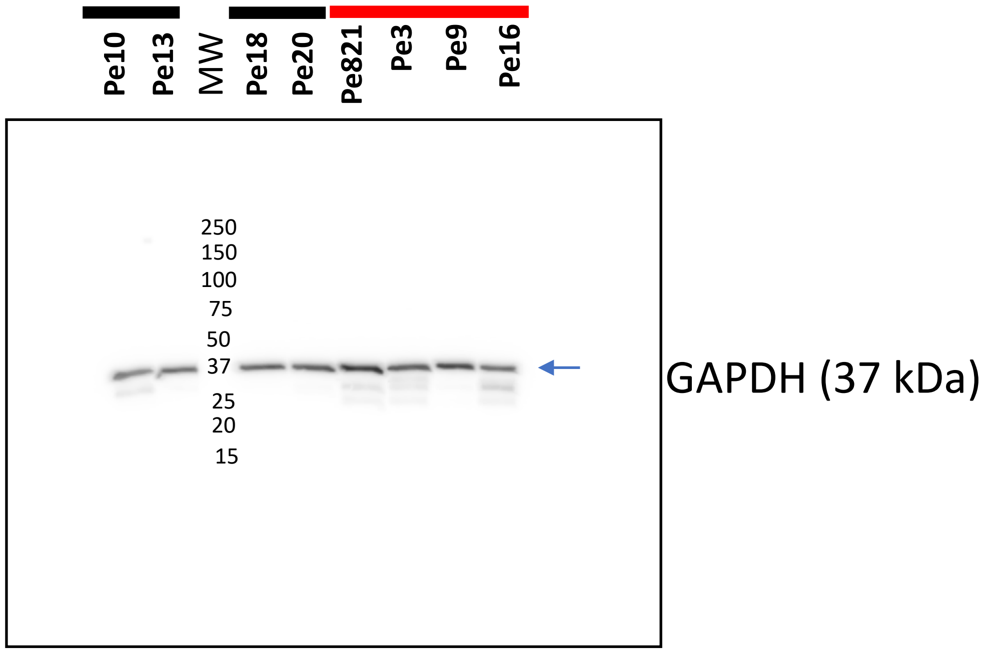

Immunoblot GAPDH
