# Supplementary material for: Transcriptomic, Proteomic, and Genomic Mutational Fraction Differences Based on HPV Status Observed in Patient-Derived Xenograft Models of Penile Squamous Cell Carcinoma
Source: Cancers (Basel). 2024 Mar 6;16(5):1066. doi: 10.3390/cancers16051066 (PMC10930474; doi:10.3390/cancers16051066)
Supplement: Supplementary file 1 [file cancers-16-01066-s001.zip › tables and figures.docx]

Transcriptomic, Proteomic, and Genomic Mutational Fraction Differences Based on HPV Status Observed in Patient-Derived Xenograft Models of Penile Squamous Cell Carcinoma

Niki M. Zacharias ^1,2,^*^,†^, Luis Segarra ^1,2,†^, Keiko Akagi ^3^, Natalie Wall Fowlkes ^4^, Huiqin Chen ^5^, Angelita Alaniz ^6^, Carolyn de la Cerda ^7^, Pedro Pesquera ^1^, Yuanxin Xi ^8^, Jing Wang ^8^, Jad Chahoud ^9^, Xin Lu ^10^, Priya Rao ^11^, Magaly Martinez-Ferrer ^12^ and Curtis A. Pettaway ^1,^*

^1^ Department of Urology, University of Texas MD Anderson Cancer Center, Houston, TX 77030, USA;
lasegarra@mdanderson.org (L.S.); pipesquera@mdanderson.org (P.P.)

^2^ MD Anderson UTHealth Graduate School, Houston, TX 77030, USA

^3^ Department of Thoracic Head & Neck Medical Oncology, MD Anderson Cancer Center,
Houston, TX 77030, USA; kakagi@mdanderson.org

^4^ Department of Veterinary Medicine & Surgery, MD Anderson Cancer Center, Houston, TX 77030, USA; nwfowlkes@mdanderson.org

^5^ Biostatistics, University of Texas MD Anderson Cancer Center, Houston, TX 77030, USA;
hchen1@mdanderson.org

^6^ Center for Health Promotion and Prevention Research, University of Texas Health Science Center
at Houston, Houston, TX 77030, USA; angelita.alaniz@uth.tmc.edu

^7^ Department of Surgical Oncology, MD Anderson Cancer Center, Houston, TX 77030, USA;
cade1@mdanderson.org

^8^ Department of Bioinformatics and Computational Biology, MD Anderson Cancer Center,
Houston, TX 77030, USA; yxi@mdanderson.org (Y.X.); jingwang@mdanderson.org (J.W.)

^9^ Department of Genitourinary Oncology, H. Lee Moffitt Cancer Center and Research Institute,
Tampa, FL 33612, USA; jad.chahoud@moffitt.org

^10^ Department of Biological Sciences, University of Notre Dame, Norte Dame, IN 46556, USA; xlu@nd.edu

^11^ Department of Pathology, University of Texas MD Anderson Cancer Center, Houston, TX 77030, USA; prao@mdanderson.org

^12^ Department of Pharmaceutical Sciences, University of Puerto Rico Medical Sciences Campus & Cancer Biology, UPR Comprehensive Cancer Center, San Juan, PR 00936, USA; magaly.martinez@upr.edu

***** Correspondence: nmzacharias@mdanderson.org (N.M.Z.); cpettawa@mdanderson.org (C.A.P.);
Tel.: +01-713-792-5226 (N.M.Z.); +01-713-792-3250 (C.A.P.)

^†^ These authors contributed equally to this work.

| ID | SX | Age | Ethnicity | Tumor Site | Histology SCC | p16 status | Pathological Staging | Grade | LVI | PNI | Rec. | Neoadjuvant Treatment | Engraftment P1 | Engraftment P3 | Current Status |
| --- | --- | --- | --- | --- | --- | --- | --- | --- | --- | --- | --- | --- | --- | --- | --- |
| Pe821 | RP | 68 | Cau | P | BSCC | Neg | rpT4NxM1 | 3 | Yes | No | Yes | Yes | Yes | Yes | LOF |
| Pe1 | ILND | 76 | AA | LN | SCC | Not Tested | pT2N2 | 1 | Yes | No | No | Yes | No | No | NED |
| Pe2 | WLE | 75 | Cau | P | SCC | Not Tested | pT1bNx | 3 | No | No | No | No | No | No | LOF |
| Pe3 | RP | 67 | Cau | P | BSCC | Pos | rpT2Nx | 3 | Yes | Yes | Yes | No | Yes | Yes | NED |
| Pe5 | PP | 69 | HL | P | SCC | Not Tested | pT2Nx | 1 | No | No | No | No | No | No | AWD |
| Pe6 | RP | 70 | HL | P | SCC | Not Tested | pT3Nx | 2 | Yes | Yes | No | No | No | No | LOF |
| Pe7* | ILND | 67 | Cau | LN |  | Neg | ypT0N0M0 |  |  |  | Yes | Yes | Yes | No | NED |
| Pe8 | RP | 74 | AA | P | VSCC | Neg | pT3Nx | 2 | No | No | No | No | Yes | No | NED |
| Pe9 | ILND | 33 | HL | LN | SCC | Pos | pT3N3Mx | 3 | No | Yes | Yes | Yes | Yes | Yes | DOC |
| Pe10 | RP | 80 | HL | P | SCC | Neg | pT3N3Mx | 3 | Yes | Yes | No | Yes | Yes | Yes | DOC |
| Pe11 | RP | 61 | HL | P | SCC | Neg | pT3Nx | 1 | No | No | Yes | No | No | No | LOF |
| Pe12* | RP | 85 | Cau | P | PSCC, BSCC |  | ypT0N0M0 | 1 | No | No | No | Yes | No | No | DOC |
| Pe13 | PP | 67 | HL | P | SCC | Neg | pT3Nx | 3 | No | Yes | No | No | Yes | Yes | LOF |
| Pe14 | PP | 70 | HL | P | Other | Neg | pT2Nx | 1 | No | No | No | No | No | No | NED |
| Pe16 | PP | 82 | Cau | P | BSCC | Pos | pT3NNo | 3 | Yes | Yes | No | No | Yes | Yes | NED |
| Pe17* | PP | 72 | Cau | P |  |  | ypT0N0M0 |  |  |  | No | Yes | No | No | NED |
| Pe18 | PP | 62 | HL | P | SCC | Neg | pT3pN3 | 3 | Yes | Yes | No | No | Yes | Yes | NED |
| Pe19 | PP | 71 | Cau | P | PSCC, BSCC | Neg | pT1aNx | 2 | No | No | No | No | No | No | NED |
| Pe20 | RP | 72 | HL | P | SCC | Neg | pT3Nx | 1 | No | No | No | No | Yes | Yes | AWD |

**Table S1**. Clinical Variables associated with tumor tissue taken from 19 patients. Abbreviations: SX (surgery type), PP (partial penectomy), RP (radical penectomy), ILND (inguinal lymph node dissection), WLE (wide local excision), Cau (Caucasian), HL (Hispanic/Latino), P (penis), LN (lymph node), SCC (squamous cell carcinoma), BSCC (basaloid SCC), VSCC (verrucous SCC), PSCC (papillary SCC),LVI (lympho-vascular invasion, PNI (perineural invasion) Rec. (Recurrence), LOF (lost to follow-up), NED (no evidence of disease), DOC (died of other cause), AWD (alive with disease). *Tissue samples from Pe7, Pe12, and Pe17 were obtained from resections performed post-chemotherapy, and upon review by pathology, no tumor was present.

| Covariate | Status | Initial Engraftment (P1) | | | 3^rd^ Passage Engraftment (P3) | | | 4/5^th^ Passage Engraftment (P4/P5) | | |
| --- | --- | --- | --- | --- | --- | --- | --- | --- | --- | --- |
|  |  | No | Yes | p-value | No | Yes | p-value | No | Yes | p-value |
| Age at diagnosis (mean + SD) |  | 72.1 (4.5) | 67.2 (13.5) | 0.306 | 71.8 (6.0) | 66.4 (15.1) | 0.341 | 71.1 (6.4) | 67.0 (16.2) | 0.703 |
| Ethnicity | White | 4 | 4 | 0.774 | 5 | 3 | 0.554 | 5 | 3 | 0.827 |
|  | African American | 1 | 1 |  | 2 | 0 |  | 2 | 0 |  |
|  | Latino | 4 | 5 |  | 4 | 5 |  | 5 | 4 |  |
| Tissue site | Lymph Node | 1 | 2 | 1.000 | 2 | 1 | 1.000 | 2 | 1 | 1.000 |
|  | Penis | 8 | 8 |  | 9 | 7 |  | 10 | 6 |  |
| PNI | Yes | 1 | 6 | 0.057 | 1 | 6 | 0.006 | 2 | 5 | 0.045 |
|  | No | 8 | 4 |  | 10 | 2 |  | 10 | 2 |  |
| LVI | Yes | 2 | 5 | 0.350 | 2 | 5 | 0.074 | 3 | 4 | 0.326 |
|  | No | 7 | 5 |  | 9 | 3 |  | 9 | 3 |  |
| Grade | 0* | 1 | 1 | 0.036 | 2 | 0 | 0.005 | 2 | 0 | 0.033 |
|  | 1 | 5 | 1 |  | 5 | 1 |  | 5 | 1 |  |
|  | 2 | 2 | 1 |  | 3 | 0 |  | 3 | 0 |  |
|  | 3 | 1 | 7 |  | 1 | 7 |  | 2 | 6 |  |
| Grade | < 3 | 8 | 3 | 0.020 | 10 | 1 | 0.001 | 10 | 1 | 0.006 |
|  | = 3 | 1 | 7 |  | 1 | 7 |  | 2 | 6 |  |
| T stage | 0* | 2 | 1 | 0.121 | 3 | 0 | 0.095 | 3 | 0 | 0.247 |
|  | 1 | 2 | 0 |  | 2 | 0 |  | 2 | 0 |  |
|  | 2 | 3 | 1 |  | 3 | 1 |  | 3 | 1 |  |
|  | 3 | 2 | 7 |  | 3 | 6 |  | 4 | 5 |  |
|  | 4 | 0 | 1 |  | 0 | 1 |  | 0 | 1 |  |
| T stage | < 3 | 7 | 2 | 0.023 | 8 | 1 | 0.020 | 7 | 1 | 0.057 |
|  | ≥ 3 | 2 | 8 |  | 3 | 7 |  | 4 | 6 |  |
| N stage | 0 | 2 | 3 | 0.609 | 3 | 2 | 0.392 | 3 | 2 | 0.247 |
|  | 1 | 6 | 4 |  | 7 | 3 |  | 7 | 3 |  |
|  | 2 | 1 | 3 |  | 1 | 3 |  | 2 | 2 |  |
| N stage | < 1 | 2 | 3 | 1.000 | 3 | 2 | 1.000 | 3 | 2 | 1.000 |
|  | ≥ 1 | 7 | 7 |  | 8 | 6 |  | 9 | 5 |  |
| Recurrence | Yes | 1 | 4 | 0.303 | 2 | 3 | 0.603 | 2 | 3 |  |
|  | No | 8 | 6 |  | 9 | 5 |  | 10 | 4 |  |
| Neoadjuvant Treatment | Yes | 3 | 4 | 1.000 | 4 | 3 | 1.000 | 4 | 3 | 1.000 |
|  | No | 6 | 6 |  | 7 | 5 |  | 8 | 4 |  |

**Table S2.** Statistical analysis of clinical characteristics of patients and tumors and successful engraftment in passage 1 (PI), passage 2 (P2), and passage 4/5 (P4/5). All clinical characteristics that were statistically significant (*p* < 0.05) in engraftment success are highlighted in red. *For Pe7, Pe12, Pe17, tumor was not present based on histology and therefore were given a grade and tumor stage of 0 for statistical analysis.


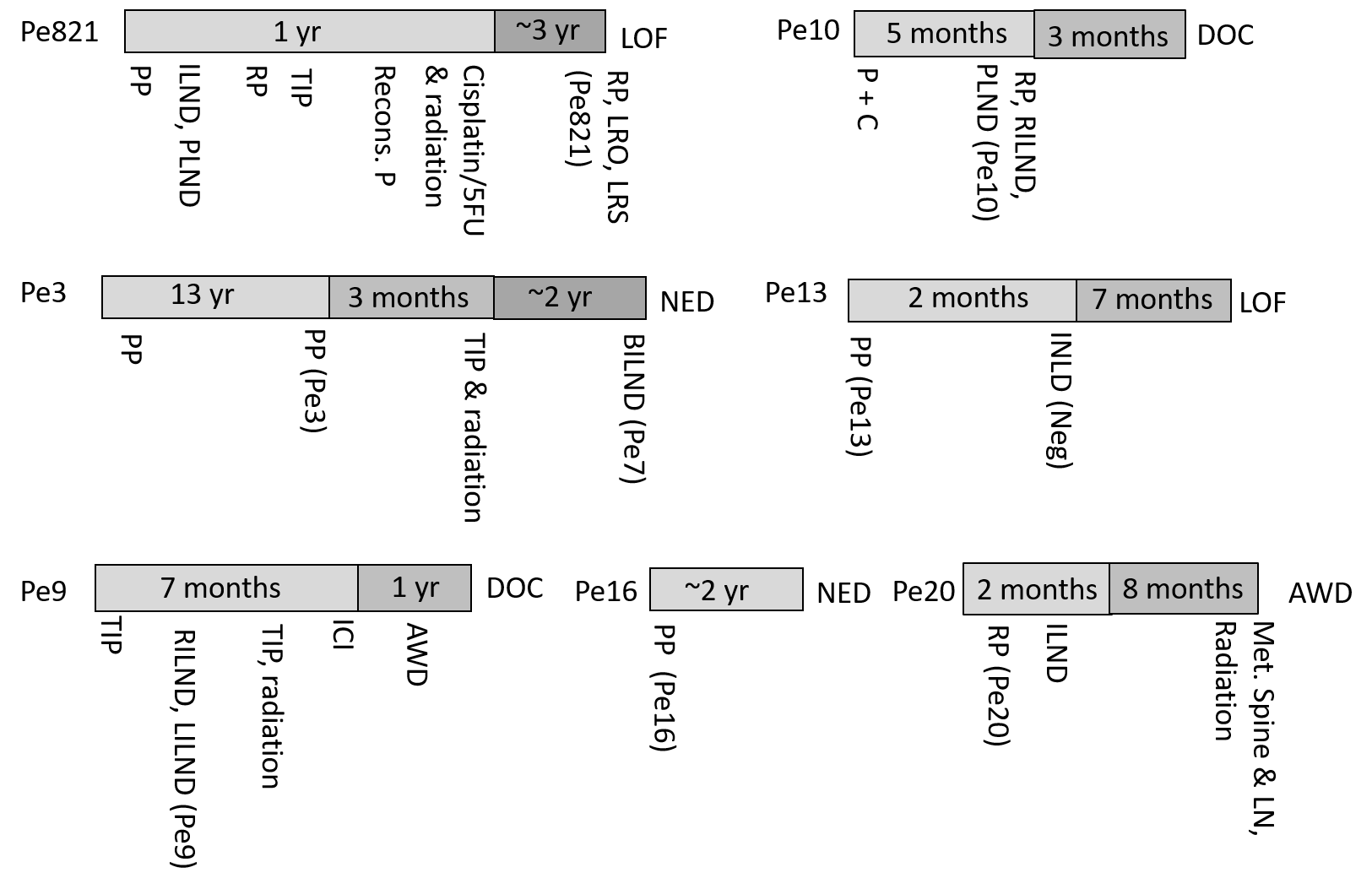


**Figure S1.** Graphical depiction of clinical treatment patients received and what resected tissue was used to generate models: XPe821, XPe3, XPe7, XPe9, XPe10, XPe13, XPe16, and XPe20. Abbreviations: PP (partial penectomy), RP (radical penectomy), Recons. P. (reconstruction of the penis), ICI (immune checkpoint inhibitor), TIP (4x cycle of TIP therapy), P + C (paclitaxel + carboplatin), INLD (inguinal lymph node dissection), PLND (pelvic lymph node dissection), LRO (left radical orchiectomy), LRS (left radical scrotectomy), BILND (bilateral inguinal lymph node dissection), RILND (right inguinal lymph node dissection), LILND (left inguinal lymph node dissection) , Met (metastasis), LN (lymph nodes), DOC (died of other cause), NED (no evidence of disease), LOF (lost to follow-up), AWD (alive with disease). Both DOCs were from COVID-19.


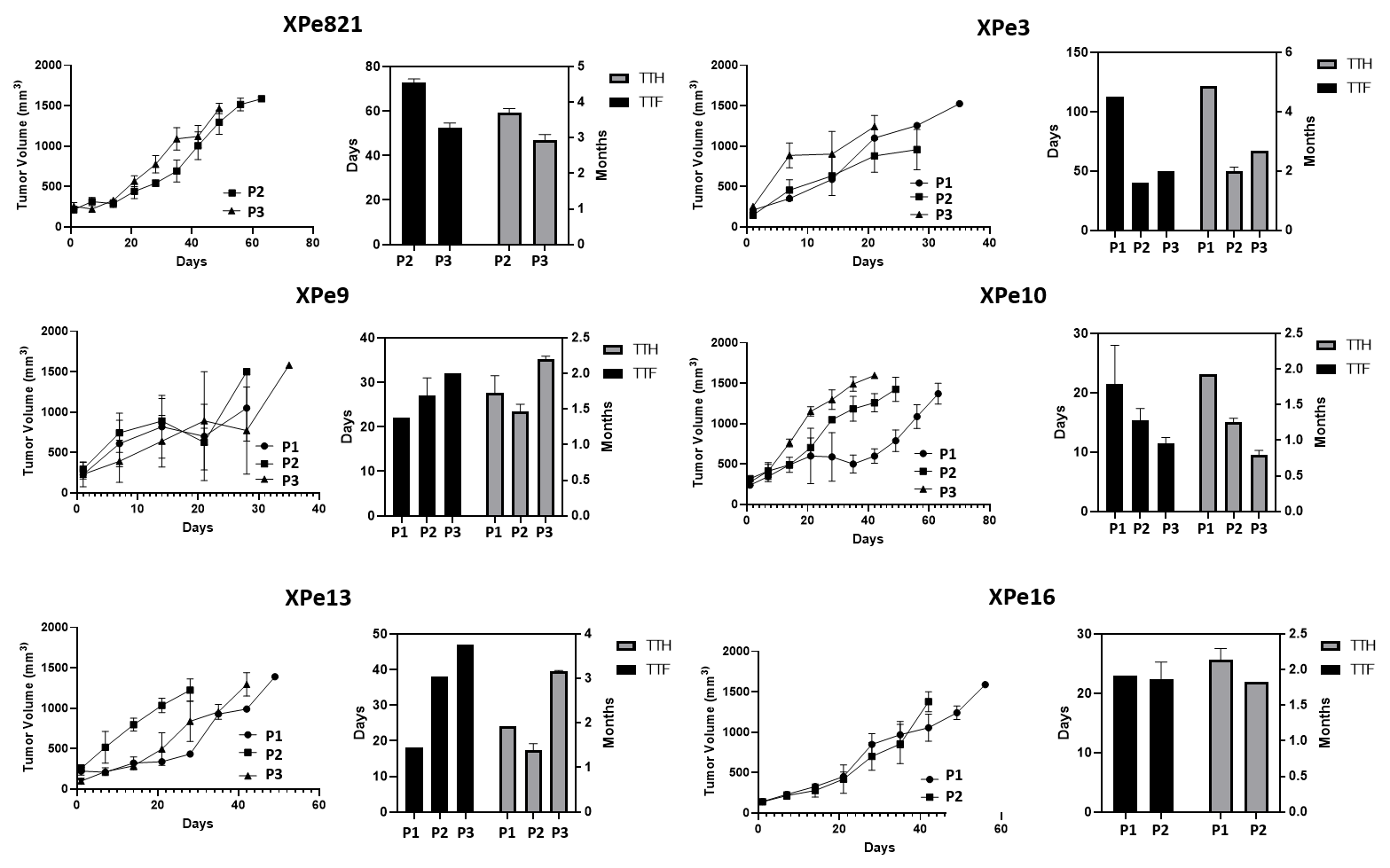


**Figure S2.** Variability is observed in tumor growth curves, time to tumor formation (TTF), and time to tumor harvest (TTH) between models and between passages for 6 models. Time to tumor formation (TTF) was defined as the time in days to first palpable tumor. While time to tumor harvest (TTH) was defined as the time in months to collection of the tumor after reaching maximum tumor volume (~1500m^3^). For each model, a tumor growth chart for the first two to the three passages (P1, P2, P3) is shown, TTF given in days is shown on left y-axis of the bar charts, and TTH is shown in months on the right y-axis of the bar charts.

|  | XPe7 | | XPe8 | | XPe18 | |
| --- | --- | --- | --- | --- | --- | --- |
| Loci | Donor | P1 | Donor | P1 | Donor | P1 |
| CSF1PO | 12 | 12 | 8,10 | 8,10 | 11, 12 | 12 |
| D13S317 | 9,11 | 9,11 | 11 | 11 | 12, 14 | 12, 14 |
| D16S539 | 9 | 9 | 11,13 | 11,13 | 11, 13 | 11, 13 |
| D18S51 | 12,13 | 12,13 | 17,18,19 | 17,18,19 | 13 | 13 |
| D21S11 | 27,33.2 | 27,33.2 | 30,31 | 30,31 | 28, 31.2 | 28, 31.2 |
| D5S818 | 11,13 | 11,13 | 11,12 | 11,12 | 9, 12 | 12 |
| D7S820 | 10,12 | 10,12 | 11,12 | 11,12 | 10, 12 | 10, 12 |
| D8S1179 | 13,16 | 13,16 | 12 | 12 | 16 | 16 |

**Table S3.** STR fingerprinting comparing donor tissue to PDX passage 1 (P1) of models XPe7, XPe8, and XPe18.

| **Gene Name** | **Protein Name in RPPA*** | **Full Protein Name** | **FDR Adjusted *p*-value in Patient RPPA** | **FDR Adjusted *p*-value in PDX RPPA** |
| --- | --- | --- | --- | --- |
| *CDKN2A* |  | Cyclin-dependent kinase inhibitor 2A | 0.00085 | 3.3E-09 |
| *STAT5A* |  | Signal transducer and activator of transcription 5A | 0.043 | 0.041 |
| *NOTCH1* |  | Neurogenic locus notch homolog protein 1 | 0.049 | 0.0045 |
| *MSH2* |  | DNA mismatch repair protein Msh2 | 0.0018 | 0.0069 |
| *AXL* |  | Tyrosine-protein kinase receptor UFO | 0.040 | 3.7E-5 |
| *TRIM28* | KAP1 | Tripartite motif-containing 28, transcriptional intermediary factor 1β, or KAP1 | 0.031 | 0.0065 |
| *RAD23A* |  | UV excision repair protein RAD23 homolog A | 0.045 | 0.030 |
| *RRM2* |  | Ribonucleoside-diphosphate reductase subunit M2 | 0.040 | 0.0013 |
| *IGF1R* | IGF-Rb | Insulin-like growth factor 1 (IGF-1) receptor | 0.032 | 0.00017 |
| *CLDN7* | Claudin-7 | Claudin-7 | 0.0063 | 0.0023 |
| *MSH6* |  | DNA mismatch repair protein MSH6 | 0.0044 | 0.028 |
| *CDC25C* |  | Cell division cycle 25C | 0.031 | 3.1E-5 |
| *ATM* | ATM-pS1981 (phosphorylated) | ATM serine/threonine kinase | 0.037 | 0.0084 |
| *ESR1* | ER α | Estrogen receptor alpha | 0.012 | 0.0029 |
| *HSPD1* | HSP60 | chaperonins (heat shock protein 1) | 0.048 | 0.0014 |
| *SGK1* |  | serum and glucocorticoid-regulated kinase 1 | 0.030 | 0.023 |
| *G6PD* |  | Glucose-6-phosphate dehydrogenase | 0.011 | 0.0066 |
| *IL6* |  | Interleukin 6 | 0.023 | 0.021 |

*If different from gene name

**Table S4**. The 18 proteins found to be differentially expressed based on HPV status in both RPPA data sets. The table gives the gene names, proteins names found in RPPA analysis if different from gene name, the full protein name, and FDR Adjusted *p*-value for each protein in the patient and the PDX data set.


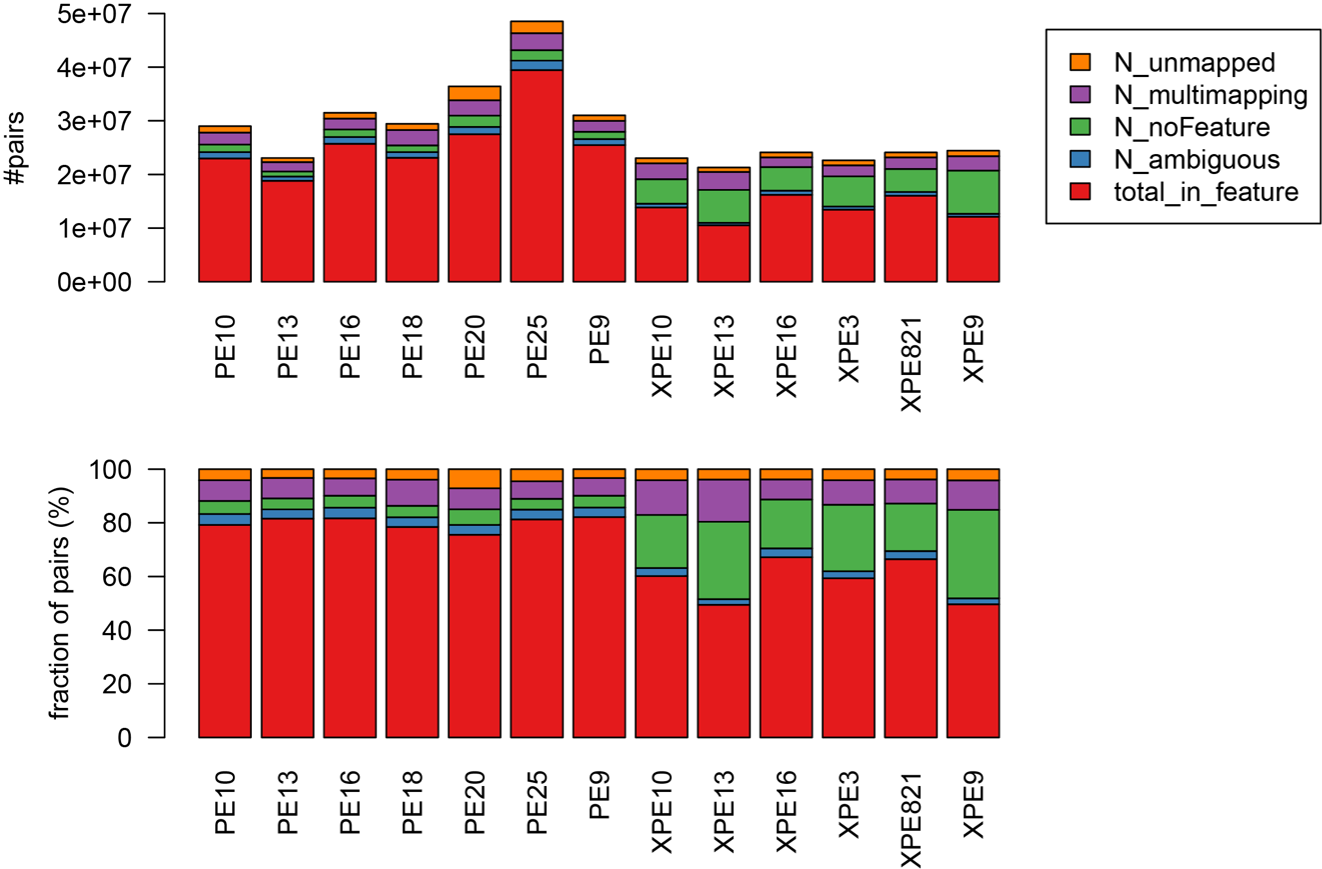


**Figure S3.** Summary of RNASeq data. In patient tumor samples (Pe10, Pe13, Pe16, Pe18, Pe20, Pe25, and Pe9) approximately 80% of reads are aligned to transcripts unique for tumor samples (red) while in xenograft samples (XPe10, XPe13, XPe16, XPe3, XPe821, and XPe9) are partially contaminated with mouse transcript. Green (N_noFeature) are reads aligned to mouse genome.


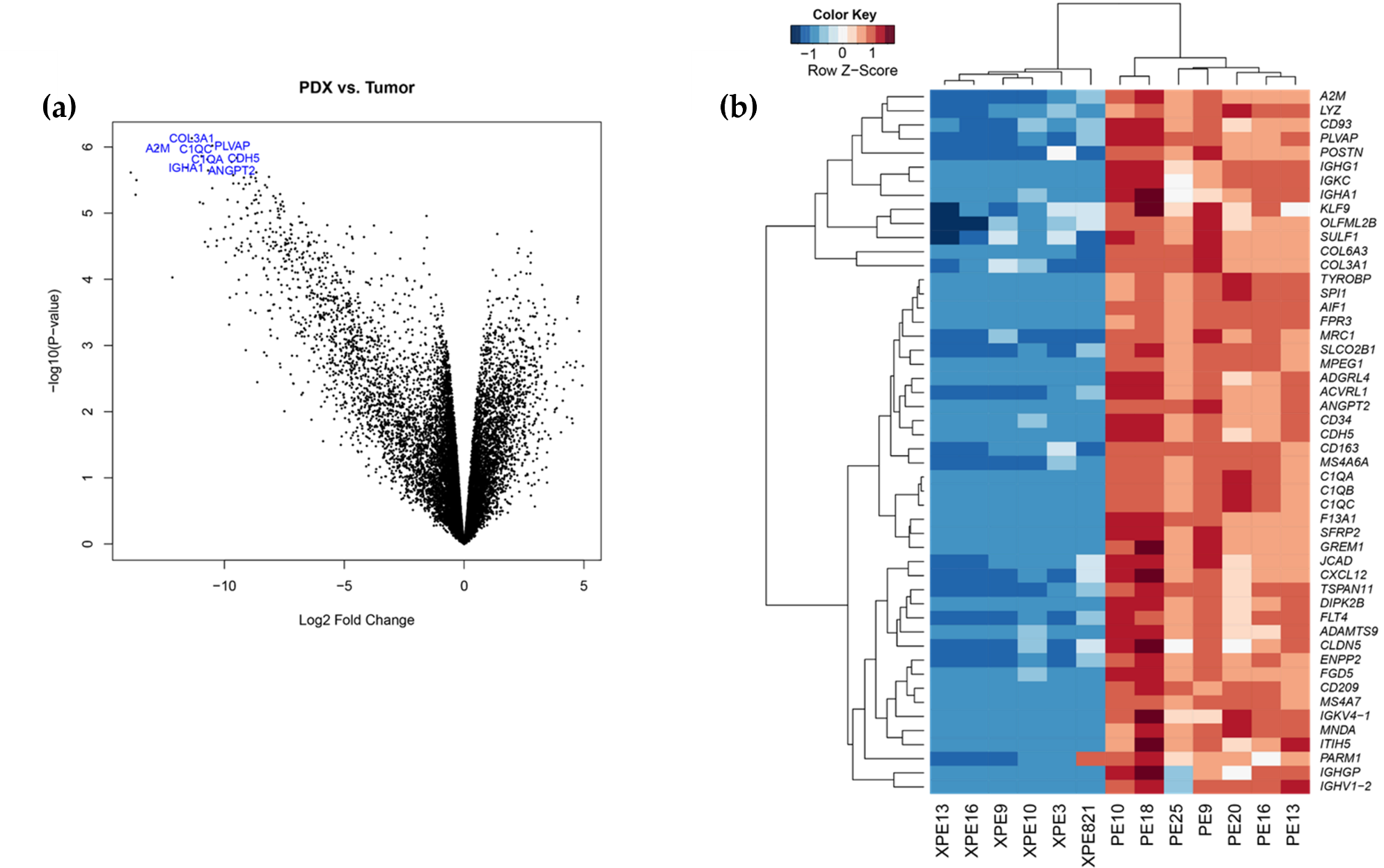


**Figure S4.** RNASeq analysis of differentially expressed genes in PDX tissue (labeled with XPe) and patient tissue (labeled Pe). 1783 genes are differentially expressed after multiple testing correction (FDR adjusted *p* < 0.05).

| **GO.ID** | **Biological Process** | **Kolmogorov-Smirnov test**  ***p*-value** |
| --- | --- | --- |
| GO:0002449 | Lymphocyte mediated immunity | < 1e-30 |
| GO:0002460 | Adaptive immune response based on somatic recombination of immune receptors built from immunoglobulin superfamily domains | < 1e-30 |
| GO:0006909 | Phagocytosis | < 1e-30 |
| GO:0042113 | B cell activation | < 1e-30 |
| GO:0042742 | Defense response to bacterium | < 1e-30 |
| GO:0006959 | Humoral immune response | 2.20E-16 |
| GO:0050851 | Antigen receptor-mediated signaling pathway | 1.10E-15 |
| GO:0002250 | Adaptive immune response | 1.30E-15 |
| GO:0050900 | Leukocyte migration | 3.20E-15 |
| GO:0060326 | Cell chemotaxis | 2.30E-13 |
| GO:0002683 | Negative regulation of immune system process | 5.10E-13 |
| GO:0007155 | Cell adhesion | 8.40E-13 |
| GO:0051251 | Positive regulation of lymphocyte activation | 2.10E-12 |
| GO:0050727 | Regulation of inflammatory response | 5.30E-12 |
| GO:0006897 | Endocytosis | 1.30E-11 |
| GO:0050670 | Regulation of lymphocyte proliferation | 1.50E-11 |
| GO:0030198 | Extracellular matrix organization | 5.20E-11 |
| GO:0051051 | Negative regulation of transport | 6.30E-11 |
| GO:0032103 | Positive regulation of response to external stimulus | 6.40E-11 |
| GO:0006954 | Inflammatory response | 7.10E-11 |

**Table S5.** Top 20 GO pathways upregulated in patient tumor tissue versus PDX tissue by RNASeq analysis.

| **GO.ID** | **Biological Process** | **Kolmogorov-Smirnov test p-value** |
| --- | --- | --- |
| GO:0006260 | DNA replication | < 1e-30 |
| GO:0051321 | Meiotic cell cycle | 6.40E-14 |
| GO:0051301 | Cell division | 1.80E-13 |
| GO:0006310 | DNA recombination | 9.20E-13 |
| GO:0051276 | Chromosome organization | 1.20E-12 |
| GO:0140014 | Mitotic nuclear division | 1.10E-11 |
| GO:0000819 | Sister chromatid segregation | 1.10E-11 |
| GO:0000226 | Microtubule cytoskeleton organization | 1.90E-10 |
| GO:0006302 | Double-strand break repair | 1.90E-10 |
| GO:1901988 | Negative regulation of cell cycle phase transition | 6.50E-09 |
| GO:0000280 | Nuclear division | 8.00E-09 |
| GO:1901990 | Regulation of mitotic cell cycle phase transition | 3.40E-08 |
| GO:0006281 | DNA repair | 5.00E-08 |
| GO:0090068 | Positive regulation of cell cycle process | 2.50E-07 |
| GO:0045930 | Negative regulation of mitotic cell cycle | 2.80E-07 |
| GO:0098813 | Nuclear chromosome segregation | 6.60E-06 |
| GO:1903047 | Mitotic cell cycle process | 1.80E-05 |
| GO:0033044 | Regulation of chromosome organization | 2.50E-05 |
| GO:0140694 | Non-membrane-bounded organelle assembly | 2.60E-05 |

**Table S6.** Gene set enrichment analysis using GO ontology terms for differentially expressed genes based on HPV status.

| Coding Change | **Pe3** | **XPe3** | **Pe9** | **XPe9** | **Pe10** | **XPe10** | **Pe13** | **XPe13** | **Pe16** | **XPe16** | **Pe20** | **XPe20** | **Pe821** | **XPe821** |
| --- | --- | --- | --- | --- | --- | --- | --- | --- | --- | --- | --- | --- | --- | --- |
| Frameshift deletion | 0 | 0 | 4 | 4 | 1 | 1 | 6 | 8 | 0 | 0 | 78 | 133 | 3 | 4 |
| Frameshift insertion | 1 | 0 | 0 | 4 | 2 | 2 | 2 | 2 | 0 | 1 | 11 | 19 | 1 | 2 |
| Non-frameshift deletion | 1 | 1 | 0 | 0 | 0 | 1 | 5 | 4 | 1 | 1 | 5 | 9 | 0 | 1 |
| Non-frameshift insertion | 0 | 0 | 0 | 0 | 0 | 0 | 7 | 8 | 0 | 1 | 1 | 1 | 0 | 0 |
| Nonsynonymous SNV | 25 | 37 | 48 | 79 | 36 | 47 | 247 | 253 | 20 | 26 | 114 | 193 | 69 | 87 |
| Splicing | 0 | 0 | 1 | 2 | 1 | 3 | 9 | 15 | 1 | 1 | 4 | 4 | 1 | 1 |
| Stopgain | 1 | 1 | 3 | 3 | 1 | 2 | 16 | 14 | 1 | 1 | 8 | 10 | 8 | 10 |
| Stoploss | 0 | 0 | 0 | 0 | 0 | 0 | 1 | 1 | 0 | 0 | 0 | 0 | 0 | 0 |

**Table S7.** Number of somatic variants with coding change consequences in both patient tumor sample (Pe) and in the PDX tissue samples (XPe). SNV stands for single nucleotide variance.

| ID Number | Somatic Variants in Donor Tissue | Somatic Variants in PDX Tissue | Somatic variants observed in both PDX and Donor Tissue | Somatic variants observed only in Donor | Somatic variants observed only in PDX | Shared Variants / Variants in PDX only |
| --- | --- | --- | --- | --- | --- | --- |
| Pe3 | 28 | 39 | 21 | 7 | 18 | 53.85% |
| Pe9 | 56 | 92 | 36 | 20 | 56 | 39.13% |
| P10 | 41 | 56 | 35 | 6 | 21 | 62.50% |
| Pe13 | 293 | 305 | 262 | 31 | 43 | 85.90% |
| Pe16 | 23 | 31 | 20 | 3 | 11 | 64.52% |
| Pe20 | 221 | 369 | 206 | 15 | 163 | 55.83% |
| Pe821 | 82 | 105 | 59 | 23 | 46 | 56.19% |

**Table S8.** Number of somatic variants found in donor tissue, in PDX tissue, and in both tissues with the percent of shared variants between donor tissue and PDX tissue versus number of variants in PDX given.
